# An essential host dietary fatty acid promotes TcpH inhibition of TcpP proteolysis promoting virulence gene expression in *Vibrio cholerae*

**DOI:** 10.1128/mbio.00721-24

**Published:** 2024-07-03

**Authors:** Lucas M. Demey, Ritam Sinha, Victor J. DiRita

**Affiliations:** 1 Department of Microbiology & Molecular Genetics, Michigan State University, East Lansing, Michigan, USA; New York University School of Medicine, New York, New York, USA

**Keywords:** virulence, regulated intramembrane proteolysis, α-linolenic acid, detergent-resistant membrane

## Abstract

*Vibrio cholerae* is a Gram-negative gastrointestinal pathogen responsible for the diarrheal disease cholera. Expression of key virulence factors, cholera toxin and toxin-coregulated pilus, is regulated directly by ToxT and indirectly by two transmembrane transcription regulators (TTRs), ToxR and TcpP, that promote the expression of *toxT*. TcpP abundance and activity are controlled by TcpH, a single-pass transmembrane protein, which protects TcpP from a two-step proteolytic process known as regulated intramembrane proteolysis (RIP). The mechanism of TcpH-mediated protection of TcpP represents a major gap in our understanding of *V. cholerae* pathogenesis. The absence of *tcpH* leads to unimpeded degradation of TcpP *in vitro* and a colonization defect in a neonate mouse model of *V. cholerae* colonization. Here, we show that TcpH protects TcpP from RIP *via* direct interaction. We also demonstrate that α-linolenic acid, a dietary fatty acid, promotes TcpH-dependent inhibition of RIP *via* co-association of TcpP and TcpH molecules within detergent-resistant membranes (DRMs) in a mechanism requiring the TcpH transmembrane domain. Taken together, our data support a model where *V. cholerae* cells use exogenous α-linolenic acid to remodel the phospholipid bilayer *in vivo*, leading to co-association of TcpP and TcpH within DRMs where RIP of TcpP is inhibited by TcpH, thereby promoting *V. cholerae* pathogenicity.

**IMPORTANCE:**

*Vibrio cholerae* continues to pose a significant global burden on health and an alternative therapeutic approach is needed, due to evolving multidrug resistance strains. Transcription of *toxT*, stimulated by TcpP and ToxR, is essential for *V. cholerae* pathogenesis. Our results show that TcpP, one of the major regulators of *toxT* gene expression, is protected from proteolysis by TcpH, *via* direct interaction. Furthermore, we identified a gut metabolite, α-linolenic acid, that stimulates the co-association of TcpP and TcpH within detergent-resistant membranes (also known as lipid-ordered membrane domains), thereby supporting TcpH-dependent antagonism of TcpP proteolysis. Data presented here extend our knowledge of RIP, virulence gene regulation in *V. cholerae*, and, to the best of our knowledge, provides the first evidence that lipid-ordered membranes exist within *V. cholerae*. The model presented here also suggests that TTRs, common among bacteria and archaea, and co-component signal transduction systems present in *Enterobacteria*, could also be influenced similarly.

## INTRODUCTION

*Vibrio cholerae* tightly regulates the expression of its virulence factors, such as cholera toxin (CtxAB) and the toxin co-regulated pilus (TcpA-F) to reach the optimal site of infection, the crypt of intestinal villi (1–6). Transcription of these essential virulence factors is regulated by ToxT, an AraC-like transcription factor (7–10). Similarly, transcription of *toxT* is highly regulated and positively stimulated by TcpP and ToxR, two transmembrane transcription regulators (TTRs) each contain a cytoplasmic DNA-binding domain, a single transmembrane domain, and a periplasmic domain (11–14). Both ToxR and TcpP directly bind to the promoter region of *toxT*, at −180 to −60 and −55 to −37 respectively, and are required for *toxT* transcription (9, 15, 16).

TcpP is regulated *via* transcription and post-translational mechanisms (17–24). Post-translational regulation of TcpP occurs by two proteases, Tail-specific protease (Tsp) and YaeL, through a process known as Regulated Intramembrane Proteolysis (RIP) (25–27). RIP is a form of gene regulation conserved across all domains of life that allows organisms to rapidly respond to extracellular cues, commonly by liberating a transcription factor or a sigma factor, from membrane sequestration (28). Two well-characterized bacterial systems controlled by RIP mechanisms are the extracytoplasmic stress response in *E. coli* and sporulation in *Bacillus subtilis*. These systems require RIP of RseA and SpoIVFB, respectively, to release their respective sigma factors (σ^E^ and pro-σ^K^) from the membrane and stimulate gene expression (29–35). Similarly, both systems have their respective TcpH analog, RseB and BofA, which function to prevent RIP of RseA and SpoIVFB *via* different mechanisms (30, 36–41). Regulation of TcpP by this mechanism diverges from these systems because the transcription activity of TcpP is not activated by RIP but, rather, is inactivated by RIP, removing TcpP from the cytoplasmic membrane and thereby decreasing *toxT* transcription (25–27). Recent work has demonstrated that TTRs are common to both archaea and bacteria, with TTRs encompassing up to 41% of all transcription factors for certain species (42). In addition, other TTRs systems similar to TcpPH, otherwise known as co-component signal transduction systems, are common among the *Enterobacteria* and are rapidly evolving (43, 44). There has been substantial work detailing how some co-component systems respond to bile salts, but our current understanding of RIP of TcpP is limited (43–50).

Under RIP-permissive conditions *in vitro*, TcpP is sensitive to proteolysis by tail-specific protease (Tsp; site-1 protease) and subsequently by YaeL protease (site-2 protease) (25–27). RIP of TcpP is inhibited by its associated protein, TcpH, under specific *in vitro* conditions (25–27). In cells lacking TcpH, TcpP is constitutively degraded via RIP (25–27). However, the mechanism by which TcpH inhibits RIP and how TcpH-dependent RIP inhibition is modulated by extracellular stimuli remains unknown.

In this report, we provide evidence that TcpH protects TcpP from RIP *via* direct interaction. Furthermore, we explore the role of the membrane, specifically detergent-resistant and detergent-soluble membranes (DRM and DSM, respectively), in regulating TcpP-TcpH association. DRM and DSM (i.e., lipid-ordered and lipid-disordered membrane domains) are known to form in both eukaryotic and prokaryotic organisms (51–57). In prokaryotes, DRMs are small phospholipid domains that exist within both inner and outer membranes (52, 53, 57). They are composed of saturated phospholipids and hopanoids that tightly interact, resulting in a structured membrane region with low fluidity. Conversely, DSMs are enriched in unsaturated phospholipids resulting in high fluidity (51–53, 55–64).

Our model suggests that *in vivo* TcpP and TcpH preferentially associate with DRMs. This leads to enhanced inhibition of RIP by TcpH, thereby resulting in elevated TcpP levels and *toxT* transcription. We also show that utilization of exogenous α-linolenic acid, a long-chain poly-unsaturated fatty acid present *in vivo*, stimulates TcpP and TcpH association within DRMs. Data generated here support a model where, once *V. cholerae* cells enter the gastrointestinal tract, cellular uptake of α-linolenic acid results in modification of the phospholipid profile and leads to an increase in the abundance of TcpP and TcpH molecules within DRMs, thereby stimulating inhibition of RIP. Our work indicates that TcpH is also likely responsive to α-linolenic acid in many *V. cholerae* strains, due to high conservation of the TcpH transmembrane domain. The work discussed here further expands our current understanding of co-component signal transduction systems common to enteric pathogens among *Enterobacteria* and provides an example as to how the cytoplasmic membrane can modulate TTR activity, common among bacteria and archaea.

## RESULTS

### Altering the transmembrane and periplasmic domains does not disrupt TcpH activity *in vitro*

**Fig 1:**
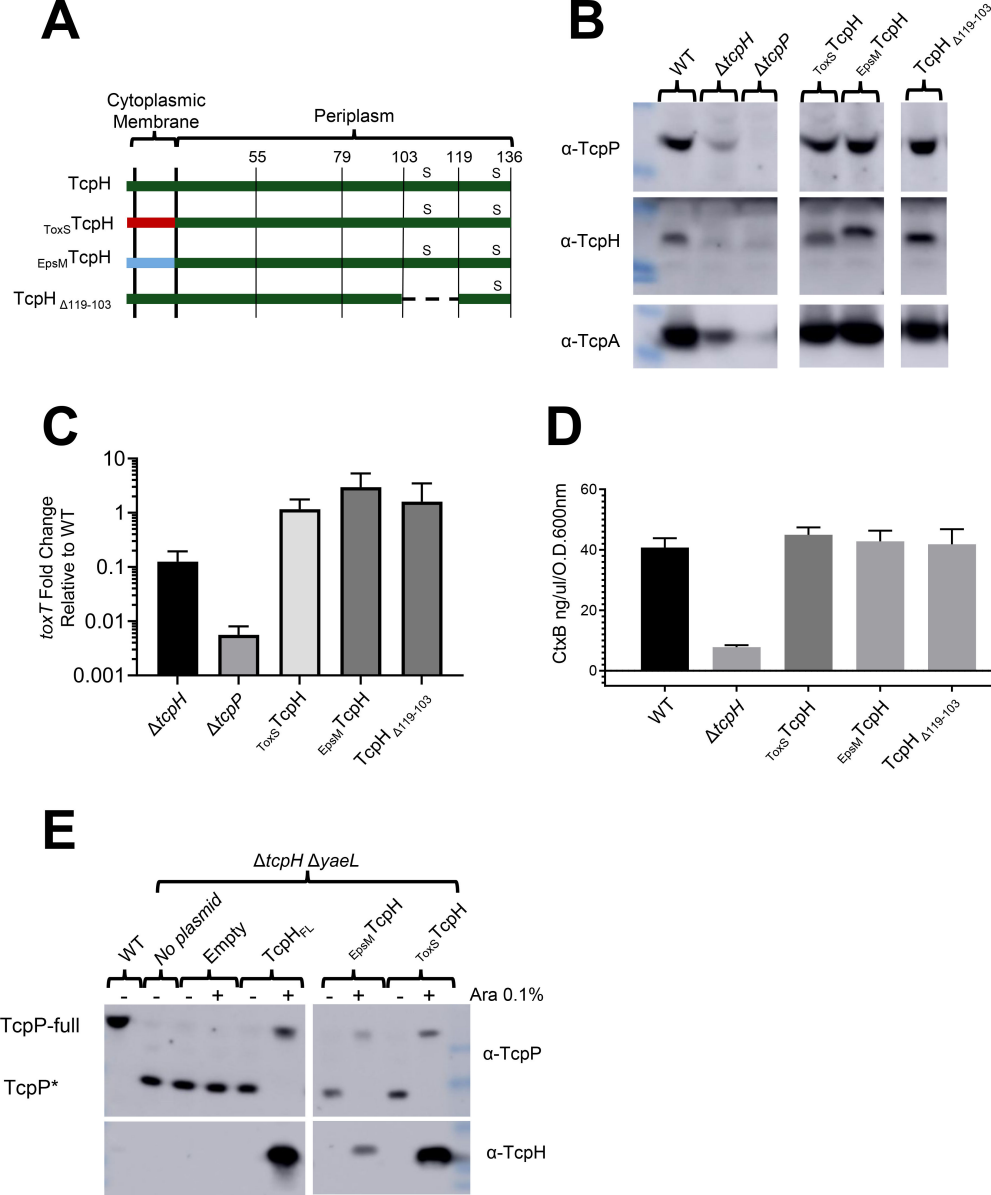
TcpH transmembrane and periplasmic constructs protect TcpP, support *toxT* expression and virulence factor production. (**A**) Diagram of TcpH transmembrane constructs (_EpsM_TcpH and _ToxS_TcpH) and periplasmic construct (TcpH_∆119-103_). TcpH has a single transmembrane domain (also a Sec signal sequence), at its N-terminus, and two periplasmic cysteine residues (C114 and C132), represented by “s.” The transmembrane domain of TcpH was replaced with the transmembrane domain of ToxS (_ToxS_TcpH) and EpsM (_EpsM_TcpH) as both ToxS and EpsM are known to be localized to the cytoplasmic membrane with similar domain topology as TcpH (65, 66). In-frame deletion of periplasmic residues is indicated by a dashed line. (**B-E**) *in vitro* characterization of TcpH transmembrane and periplasmic chromosomal constructs grown under virulence-inducing conditions. Data presented in these panels were collected from three independent experiments. (**B**) Western blots of whole-cell lysates probed with α-TcpP (top), α-TcpH (middle), and α-TcpA (bottom). (**C**) Average *toxT* transcription of TcpH variants, determined *via* ∆∆CT method. *toxT* fold change is relative to WT *V. cholerae*. (**D**) CtxB levels, measured *via* enzyme-linked immunosorbent assay, in culture supernatants collected from cultures incubated with *V. cholerae* cells cultured in virulence-inducing conditions for 24 hours. Error bars represent the standard error of the mean. See Fig. S1B for the unmodified western blots in panel B. (**C-D**) Samples from independent experiments were averaged across three technical replicates. (**E**) Western blots of spheroplast fractions (cytoplasm and cytoplasmic membrane fractions). TcpH transmembrane constructs (_ToxS_TcpH and _EpsM_TcpH) and native TcpH were expressed from pBAD18 in Δ*tcpH* Δ*yaeL* background under virulence-inducing conditions for 6 hours. All strains, excluding WT, are Δ*tcpH* Δ*yaeL*.

To identify regions within TcpH critical for its role in protecting TcpP from RIP, we constructed transmembrane (TM) domain chimeric fusions and periplasmic TcpH deletions (Peri). Two TM chimeras and one Peri deletion strain (_ToxS_TcpH, _EpsM_TcpH, and TcpH_∆103-119_, respectively) were tested, and the allele encoding each was recombined into the *V. cholerae* genome so as not to disrupt the *tcpP* coding sequence, and under normal *tcpPH* transcriptional control (Fig. 1A). Growth of the resulting strains was unaffected in comparison with wild-type *V. cholerae* in virulence inducing (Vir Ind) conditions (i.e., LB pH 6.5, 30°C) (Fig. S1A). Modification of the TcpH TM or Peri domain still supported protection of TcpP, WT *toxT* transcription, and regulated virulence factor production similar to WT TcpH and better than Δ*tcpH* (Fig. 1B through D; Fig. S1B).

While the TM TcpH supports higher levels of TcpP *in vitro* compared to Δ*tcpH*, we sought to determine whether the TM TcpH chimeras specifically inhibited RIP of TcpP. RIP of TcpP can be measured directly in a Δ*yaeL* mutant as the substrate of YaeL is a TcpP degradation intermediate (TcpP*) (27). Thus, TcpP* accumulates under RIP permissive conditions (i.e., LB pH 8.5, 37°C) or in the absence of *tcpH* in Δ*yaeL* cells (27). TcpP* lacks most of its periplasmic domain and therefore has a lower molecular weight (~17 KDa) compared to TcpP (~29 KDa), thus enabling us to determine the RIP status of TcpP *via* western blot. When TcpH is active and RIP is thereby inhibited, we observe only full-length TcpP and no TcpP*. When TcpH, _ToxS_TcpH, or _EpsM_TcpH constructs were ectopically expressed, we observed only full-length TcpP and no TcpP* (Fig. 1E). Taken together, our data indicate that _ToxS_TcpH and _EpsM_TcpH inhibit RIP of TcpP *in vitro*.

### TcpH TM domain is critical for the colonization of infant mice

**Fig 2:**
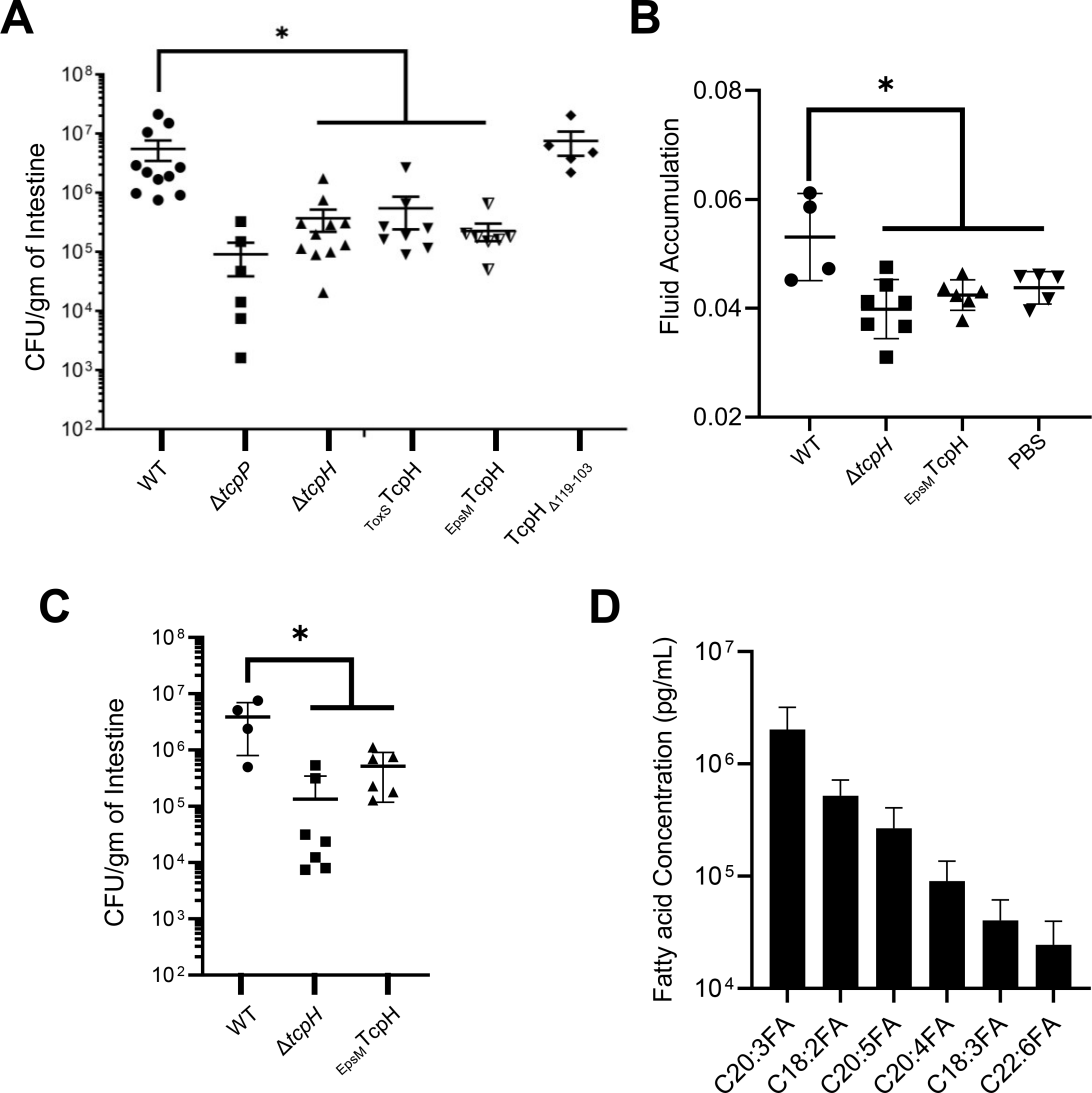
TcpH transmembrane domain is critical *in vivo*. (**A**) Colony forming units (CFUs) per gram of 3- to 6-day-old infant mouse intestine 21 hours post-infection (1 × 10^6^ inoculum dose). The horizontal line indicates the average CFU/g of intestine. These data were collected from three independent experiments with 5–11 replicates per group. (**B and C**) Fluid accumulation and CFUs per gram of infant mouse intestine 18 hours after infection (1 × 10^8^ inoculum dose). Data were collected from two independent experiments. (**D**) Representative fatty acids were identified in the lumen of infant mouse intestine, see SI Appendix Table S1 for a complete list of fatty acids identified. Data presented here are an average of 9 replicates. Error bars represent standard error of the mean in panels A–C and standard deviation of the mean in panel D. A Mann-Whitney U test (panels A and B) and a one-way ANOVA (panel C) were used to determine statistical significance. * Indicates a *P*-value less than 0.05.

The TM and Peri domain of TcpH can withstand considerable modifications and maintain function during *in vitro* experiments. Despite their wild-type activity *in vitro*, strains expressing _ToxS_TcpH, and _EpsM_TcpH colonized infant mice to levels significantly lower than the wild type, more closely resembling colonization levels of Δ*tcpH* (Fig. 2A). TcpH_∆103-119_ supported the same level of TcpH-dependent virulence gene expression *in vitro* as both _ToxS_TcpH and _EpsM_TcpH but colonized infant mice to a similar degree as wild type (Fig. 2A). As TcpA expression is critical for *V. cholerae* to colonize infant mice, we examined TcpA levels in the inocula of _ToxS_TcpH and _EpsM_TcpH used to infect infant mice. We found that _ToxS_TcpH and _EpsM_TcpH produced similar levels of TcpA compared to wild type (Fig. S2A).

We also quantified fluid accumulation (which requires a higher inoculum cell density) in infected mice, which is dependent on CtxAB synthesis. Due to no observable differences between _EpsM_TcpH and _ToxS_TcpH colonization of infant mice, _ToxS_TcpH was not included in these experiments. Mice infected with _EpsM_TcpH and Δ*tcpH* exhibited lower fluid accumulation compared to those infected with wild-type *V. cholerae,* and _EpsM_TcpH was unable to colonize infant mice to wild-type levels (Fig. 2B and C). These data support our hypothesis that _EpsM_TcpH is unable to colonize infant mice due to an inability to support virulence factor production *in vivo*.

To determine whether the presence of other microbes in the gastrointestinal tract might influence the ability of the TM TcpH strains to support pathogenicity, we cultured wild type and the TM TcpH constructs (TM and Peri) aerobically in both filter-sterilized and non-filtered mouse fecal media for 21 hours (Fig. S2B and C). Given that *V. cholerae* colonizes the small intestine and that this area of the gastrointestinal tract is relatively oxygen limited, these testing conditions are sub-optimal. However, all strains exhibited similar cell densities at the indicated time points in both filtered and non-filtered fecal media (Fig. S2B and C). While end-point cell densities of all strains were similar, those expressing _ToxS_TcpH and _EpsM_TcpH produced TcpA levels below that of wild type, although not statistically significant (Fig. S2D). Deletion of TcpH periplasmic residues had no effect on TcpA production (Fig. S2D). These data do not suggest that members of the mouse gastrointestinal microflora negatively impact the growth of the TM TcpH strains. Taken together, these data suggest that the TcpH TM domain is critical for TcpH function in the gastrointestinal tract to protect TcpP from RIP, thereby supporting downstream virulence factor production. In support of this, we also found that the TcpH TM domain is highly conserved among *V. cholerae* strains (Fig. S3). Due to its wild-type levels of colonization and ability to support wild-type levels of TcpA synthesis in mouse fecal media, we excluded the TcpH Peri deletion strain from further experiments.

### *toxT* transcription is enhanced with crude bile and is dependent on the TcpH TM domain

Data presented here and elsewhere indicate that TcpH-dependent RIP inhibition is affected by different *in vitro* and *in vivo* environmental signals and that the TM domain of TcpH is critical for that function (25–27). *Vibrio* species can use exogenous fatty acids present in bile (e.g., linoleic, linolenic, dihomo-gamma-linolenic, arachidonic, eicosapentaenoic, and docosahexaenoic acid) *via* the VolA and FadL/FadD pathways (67–71), resulting in modification of phospholipid composition (71, 72). As our data suggested that the TM domain of TcpH is important *in vivo*, we sought to determine whether there are fatty acids present in the infant mouse gastrointestinal tract that can be utilized directly by *V. cholerae*. We identified 62 different fatty acids within the infant mouse gastrointestinal tract, see Table S1. Among the 12 most abundant fatty acids, we found that linoleic (18:2), linolenic (18:3), dihomo-gamma-linolenic (20:3), arachidonic (20:4), eicosapentaenoic (20:5), and docosahexaenoic acid (22:6) are present in the infant mouse gut (Fig. 2D; Table S1). Moreover, the fatty acid profile within infant mice is similar to humans with oleic acid (18:1), linolic (18:2), α-linolenic (18:3), stearic (14:0), and palmitic acid (16:0) as the major fatty acids present (Table S1) (73) As our data demonstrate that the infant mouse gut contains fatty acids that *V. cholerae* utilizes to modify its membrane, we sought to determine whether phospholipid changes could influence TcpH-dependent inhibition of TcpP RIP. We measured *toxT* expression from a transcription reporter (pBH6119-*toxT::GFP*) in cells grown in media supplemented with Bovine Crude Bile (0.4%), which contains various fatty acids that are utilized by *V. cholerae* to remodel its membrane (71). Transcription of *toxT* from this reporter was elevated in the presence of crude bile in WT cells, which is consistent with prior studies (74), but not in cells expressing _EpsM_TcpH or _ToxS_TcpH (Fig. S4A). This suggested that native TcpH responds to changes in phospholipid composition to inhibit RIP and that the TM domain of TcpH is essential to sense and/or respond to this change. As a negative control, we measured *toxT* transcription under non-inducing conditions known to stimulate RIP of TcpP (25–27), and in these conditions, *toxT* expression was indeed reduced (Fig. S4A). In addition, we measured *toxT* expression in ∆*tcpH* cells with and without crude bile present, observing no increase in *toxT* expression (Fig. S4A). This confirms that the conditions used here do not simply promote TcpP function in the absence of TcpH.

We also measured *toxT* transcript levels directly *via* RT-qPCR with RNA isolated from wild-type cells grown in the presence of crude bile (Fig. S4B) and observed a similar increase in *toxT* transcription. Lastly, we found that cells expressing native TcpH or TcpH TM chimeras grew with similar rates in crude bile-supplemented media (Fig. S1C). Taken together, these data support a model that TcpH responds to host stimuli, specifically fatty acids or constituents of crude bile, through a mechanism requiring its native TM, and antagonizes RIP of TcpP, leading to increased *toxT* transcription.

### α-Linolenic acid enhances *toxT* expression by promoting TcpH-dependent inhibition of RIP

**Fig 3:**
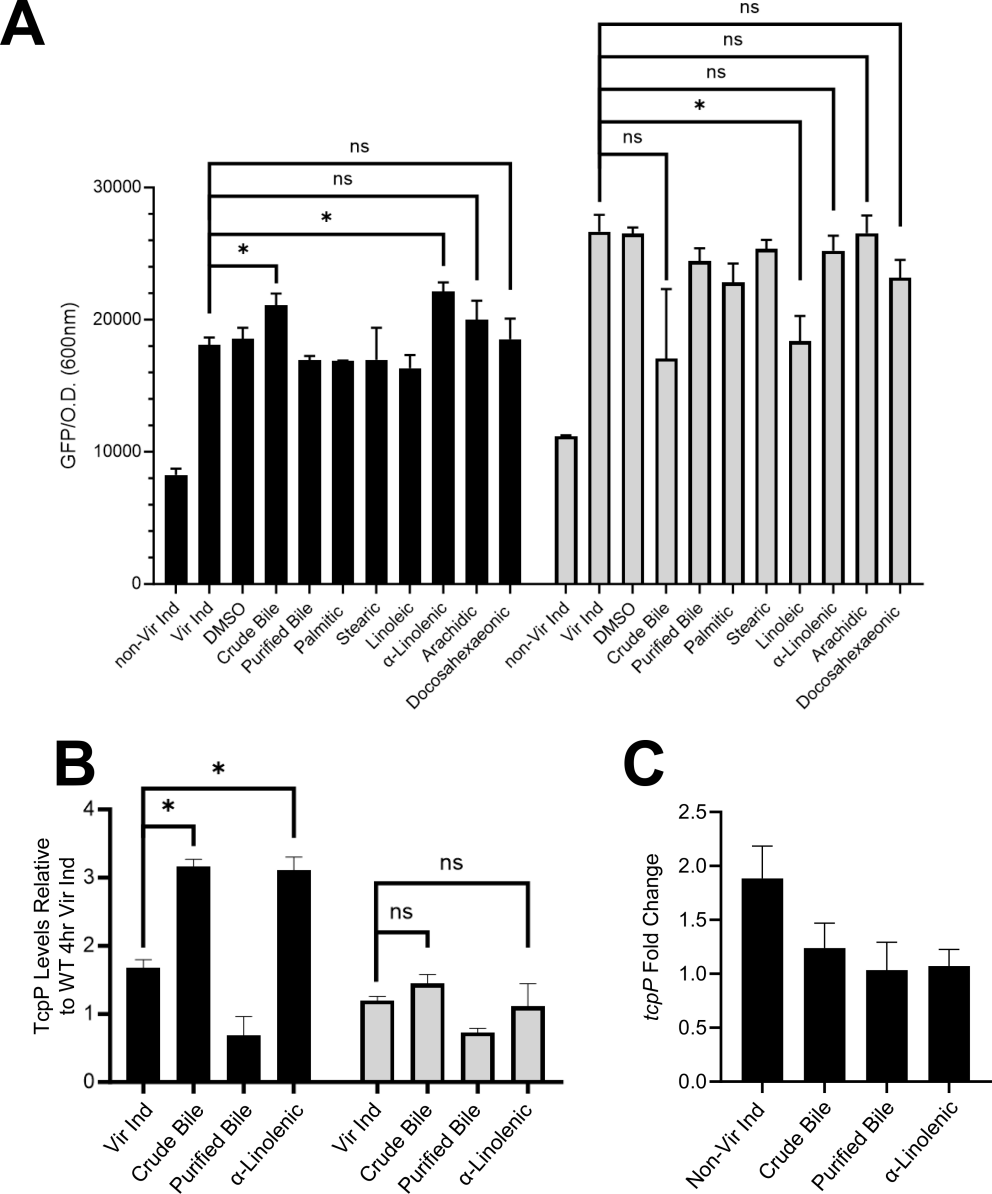
α-Linolenic acid stimulates a TcpH transmembrane-dependent increase in *toxT* transcription, elevated TcpP levels, and does not increase *tcpP* expression. (**A**) *toxT* expression in WT (black bars) and _EpsM_TcpH (gray bars) was determined using a plasmid based *toxT::GFP* transcription reporter. *toxT* transcription was determined by measuring GFP fluorescence (excitation 488 nm and emission 515 nm) and optical density (600 nm). (**B**) TcpP abundance in WT (black bars) and _EpsM_TcpH (gray bars) cells cultured for 8 hours relative to WT cells cultured under virulence inducing conditions for 4 hours. TcpP abundance was quantified *via* densitometry analysis of western blots, calculated by ImageJ. See Fig. S10B for westerns used for densitometry analysis. (**C**) *tcpP* transcription in WT *V. cholerae* cells using RT-qPCR, determined *via* ∆∆C_T_ method. *tcpP* transcription is relative to WT Vir Ind. (**A–C**) The data here are an average of three or more independent experiments, and error bars represent the standard error of the mean. (**A and B**) A one-way ANOVA was used to determine statistical significance. * Indicates a *P*-value of less than 0.05.

Crude bile is a mixture of saturated and unsaturated fatty acids, as well as bile salts (e.g., cholate and deoxycholate). To determine whether bile salts or fatty acids in crude bile were responsible for elevated *toxT* transcription in WT, we supplemented virulence-inducing media with cholate/deoxycholate (Purified Bile) (100 µM of each), palmitic acid (500 µM), stearic acid (500 µM), linoleic (500 µM), α-linolenic acid (500 µM), arachidonic acid (500 µM), and docosahexaenoic acid (500 µM). The concentration of bile and fatty acids chosen has also been used in prior studies (71). Using the *toxT*::GFP transcription reporter plasmid, we observed elevated *toxT* transcription in wild-type cells with only crude bile or α-linolenic acid present (Fig. 3A; Fig. S4A). Increased *toxT* expression with crude bile or α-linolenic acid was not observed in *∆tcpH,*_EpsM_TcpH or _ToxS_TcpH cells (Fig. S4A), demonstrating that TcpH, and its native TM domain, is still needed to inhibit RIP and TcpP is necessary to promote *toxT* transcription in the presence of these compounds. In addition to the results obtained with the *toxT::GFP* reporter, we measured *toxT* mRNA levels using RT-PCR in WT cells grown under the same conditions. Consistent with the reporter plasmid data, we observed elevated *toxT* mRNA in the presence of α-linolenic acid (~2.5 fold) (Fig. S4B). Using a *toxT::GFP* fusion, we determined that the optimal concentration for elevated expression is 50 µM linolenic acid (Fig. S4C). There was no difference in growth rate in cells expressing either native TcpH or TcpH TM chimeras when cultured with purified bile or α-linolenic acid (Fig. S1D and E). Similar concentrations of other unsaturated fatty acids (linoleic, arachidonic, and docosahexaenoic acid) did not lead to an increase in *toxT* expression (Fig. S4D).

We reasoned that enhanced *toxT* transcription in the presence of crude bile or α-linolenic acid was due to enhanced inhibition of RIP, leading, in turn, to elevated levels of TcpP. Thus, we quantified TcpP levels under Vir Ind conditions supplemented with crude bile or α-linolenic acid (Fig. 3B). TcpP levels in wild-type cells were significantly elevated in the presence of crude bile or α-linolenic acid (Fig. 3B). By contrast, growth in α-linolenic acid had no effect on TcpP levels in cells expressing _EpsM_TcpH (Fig. 3B). Loss of TcpH led to degradation of TcpP under all conditions indicating that Tsp and YaeL activity is not inhibited by the addition of crude bile or α-linolenic acid (Fig. S10B). We conclude that (i) elevated *toxT* transcription in the presence of crude bile or α-linolenic acid is due to enhanced inhibition of RIP *via* TcpH and (ii) altering the phospholipid composition of the cells with exogenous crude bile or α-linolenic acid enhances TcpH function in RIP inhibition through a mechanism that requires the native transmembrane domain.

As TcpP levels are elevated upon supplementation of crude bile or α-linolenic acid, we considered it possible that elevated *tcpP* transcription could contribute to elevated TcpP levels. One possible mechanism is that *tcpP* transcription is directly influenced by α-linolenic within the cytoplasm. Prior studies have shown that linoleic acid can rapidly diffuse into the cytoplasm of *V. cholerae* where we reasoned it might influence *tcpP* gene expression (75, 76). To determine whether *tcpP* transcription is influenced by crude bile or α-linolenic acid, we measured *tcpP* transcription in wild-type *V. cholerae* cells using both RT-qPCR and a transcription reporter, *tcpP::lacZ*. Neither crude bile nor α-linolenic acid supplementation led to increased *tcpP* transcription (Fig. 3C; Fig. S5A). These data indicate that crude bile and α-linolenic acid influence TcpP levels post-transcriptionally.

To confirm that *V. cholerae* cells incorporate α-linolenic acid into phospholipids under our growth conditions, we analyzed the fatty acid profile of phospholipids from *V. cholerae* cells cultured with and without α-linolenic acid (Fig. S5B). In the presence of α-linolenic acid, more than 80% of acyl chains within *V. cholerae* were 18:3. This is consistent with prior published data (71, 72). Furthermore, prior studies indicate that *V. cholerae* does not synthesize 18:3 fatty acids under standard laboratory conditions (77, 78) Taken together, these data suggest that *V. cholerae* cells are utilizing exogenous α-linolenic acid for phospholipid synthesis (Fig. S5B).

We next sought to determine whether WT and _EpsM_TcpH were equally capable of utilizing exogenous fatty acids. We cultured cells with cerulenin, an inhibitor of *de novo* fatty acid synthesis, or with cerulenin plus exogenous fatty acids (79–82). Cerulenin alone led to a growth defect irrespective of which form of TcpH was being expressed (Fig. S5C). The inclusion of saturated or unsaturated fatty acids restored partial growth of both WT and _EpsM_TcpH (Fig. S5C). These data indicate that WT and _EpsM_TcpH are equally capable of incorporating exogenous fatty acids into their phospholipid bilayer.

### Co-association of TcpP and TcpH with detergent-resistant membranes is required for enhanced RIP inhibition

**Fig 4:**
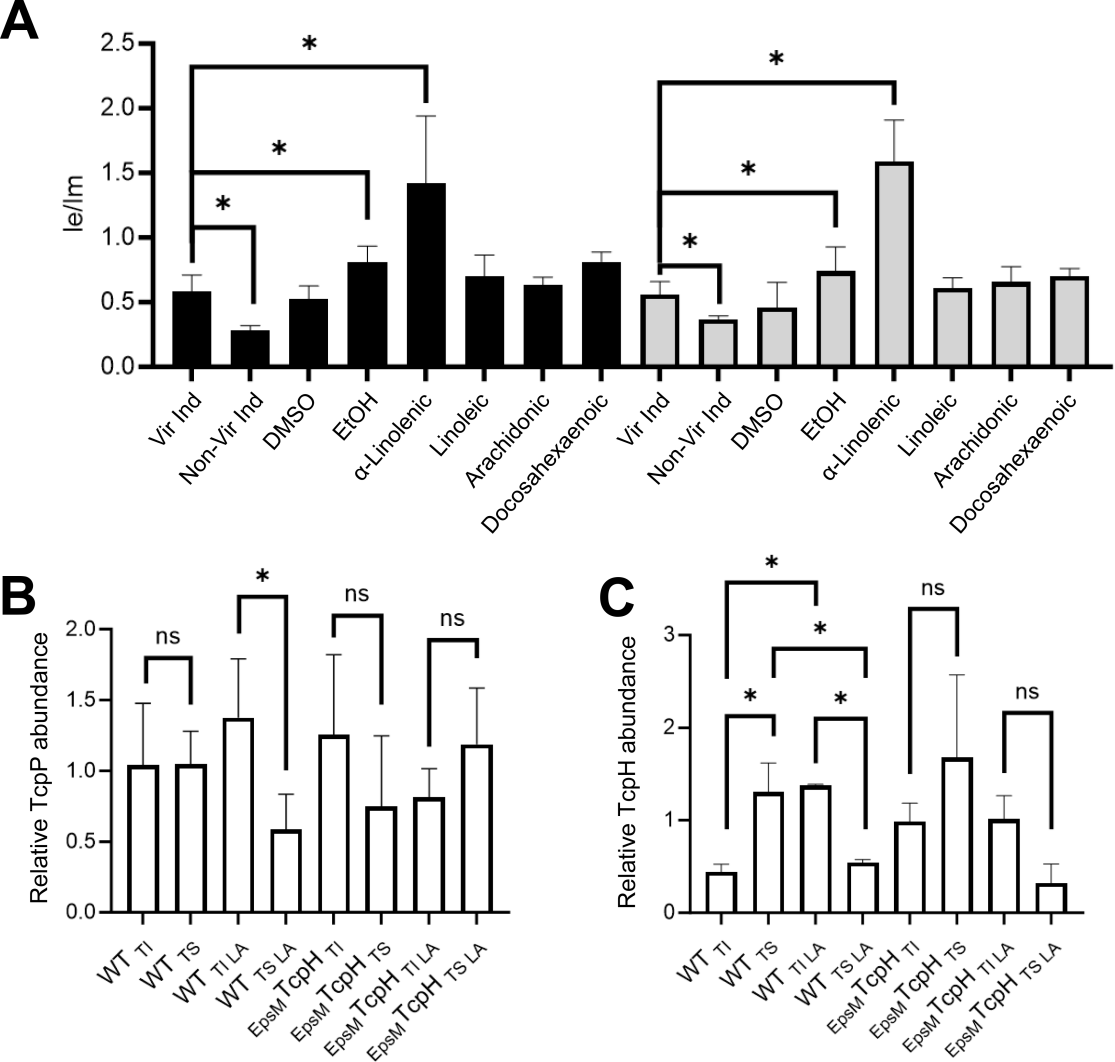
TcpP and TcpH abundance increases in detergent-resistant membranes in the presence of α-linolenic acid. (**A**) Membrane fluidity of WT (black bars) and _EpsM_TcpH (gray bars) cells, cultured with and without unsaturated fatty acids, determined by the ratio of excimer (470 nm) and monomer (400 nm) of pyrenedecanoic acid. Data were collected from three or more independent experiments. (**B**) The abundance of TcpP molecules within the Triton soluble (i.e., TS; lipid disordered) and Triton insoluble (i.e., TI; lipid ordered) fractions in WT and _EpsM_TcpH cells. (**C**) Relative abundance of TcpH and _EpsM_TcpH within the TI and TS membrane fractions. (**B-C**) Data presented here were collected from three independent experiments. TcpP and TcpH abundance were measured via densitometry using ImageJ. See Fig. S10 for representative western blots. TI and TS membrane fractions were collected by gentle freeze-thaw lysis. Cells that were cultured in α-linolenic acid (LA, 500 µM) are indicated by +. (**A–C**) Error bars represent the standard deviation. A one-way ANOVA was used to determine statistical significance. * Indicates a *P*-value less than 0.05, and ns indicates a lack of statistical significance.

Our work demonstrates that under conditions that modify phospholipid composition, TcpP levels are enhanced, and *toxT* transcription is increased. Elevated levels of TcpP are due to enhanced inhibition of RIP by TcpH rather than increased *tcpP* transcription, and this inhibitory function requires the native TcpH TM domain. In addition to α-linolenic acid, arachidonic and docosahexaenoic acid also modify phospholipid composition in *V. cholerae* (71). Despite causing similar changes to the phospholipid profile, these polyunsaturated fatty acids do not have a significant effect on *toxT* transcription (Fig. 3A; Fig. S4AD). These data indicate the phospholipid profile does not predict TcpH-dependent inhibition of RIP. Exogenous fatty acids can be utilized directly as acyl chains in *de novo* phospholipid synthesis (83, 84). Thus, while gross phospholipid composition can remain similar with supplementation of α-linolenic, arachidonic, and docosahexaenoic acid, (i.e., relative abundance of cardiolipin, phosphatidylglycerol, and phosphatidylethanolamine), the overall biophysical properties of the cytoplasmic membrane (i.e., membrane fluidity) can differ due to differences in acyl chain composition. We reasoned that the differences in observed TcpH-dependent enhanced RIP inhibition could be due to differences in the biophysical properties of the cytoplasmic membrane (e.g., membrane fluidity). To test this, we quantified membrane fluidity in WT and _EpsM_TcpH with and without exogenous fatty acids using a fluorescent lipophilic pyrene-based probe (Fig. 4A). Cells cultured with α-linolenic acid demonstrated elevated membrane fluidity, observed as a higher ratio of dimeric to monomeric pyrene probe (Fig. 4A). We did not observe a change in membrane fluidity in WT or _EpsM_TcpH cells cultured with linoleic, arachidonic, or docosahexaenoic acid (Fig. 4A).

Poly-unsaturated fatty acids (PUFA), such as omega-3 fatty acids, influence lipid-ordered membrane domains within the cytoplasmic membrane of T-cells (85, 86). Lipid-ordered membrane domains, also called lipid rafts, are regions of the membrane enriched in saturated fatty acids, cholesterol (or, in some bacterial species, hopanoids), and proteins with specific TM domain qualities (e.g., long TM domain(s) and low TM surface area) (56, 64, 87). As a result, lipid-ordered membrane domains tend to be thicker and less fluid than other areas of the membrane (55). n3-PUFA (i.e., omega-3 fatty acids) increase the size and stability of lipid-ordered membrane domains and thereby can influence membrane protein association within lipid rafts (56, 85, 86). We hypothesized that TcpP and TcpH molecules can associate within lipid-ordered membrane domains and that α-linolenic acid (an omega-3 fatty acid) supplementation increases the association of TcpP and TcpH molecules within lipid-ordered membrane domains.

Lipid-ordered membrane domains, also known as DRMs, are defined due to their insolubility in Triton X-100 (61, 88). Triton X-100 has been used in the study of both eukaryotic and prokaryotic organisms to isolate lipid-ordered and lipid-disordered membrane domains (51–56). Thus, to test our hypotheses, we used Triton X-100 to separate lipid-ordered and lipid-disordered membrane domains from cellular lysates.

Under Vir Ind conditions, TcpP and TcpH associate with Triton X-100 insoluble (TI; considered to be enriched with lipid-ordered membrane domains) and Triton X-100 soluble membrane fractions (TS; considered to be enriched with lipid-disordered membrane domains) (Fig. 4B and C). Supplementation with α-linolenic acid resulted in increases in both TcpP and TcpH in the TI fraction in WT cells (Fig. 4B and C). Like TcpH, _EpsM_TcpH is also associated with both the TI and TS membrane fractions (Fig. 4C). In contrast to WT, there was no observable increase in _EpsM_TcpH or TcpP levels in the TI fraction upon growth with α-linolenic acid in _EpsM_TcpH expressing cells (Fig. 4B and C). Surprisingly, we observed a decrease in _EpsM_TcpH abundance in the TS fraction during growth with α-linolenic acid (Fig. 4C). It is unclear why _EpsM_TcpH levels decrease in the TS fraction when α-linolenic acid is present. Our data indicate that there is a trend of lower TcpP levels in the TS fraction in _EpsM_TcpH cells cultured with α-linolenic acid (Fig. 4B; Fig. S6A and B). TcpH stability has been reported to be improved by TcpP and, thus, reduction of TcpP abundance in the TS fraction could contribute to lower _EpsM_TcpH levels in the TS fraction (89). However, it is also possible that α-linolenic acid utilization simply reduces the stability of _EpsM_TcpH in the TS fraction specifically. Regardless, it remains unclear why _EpsM_TcpH levels decrease in the TS fraction when α-linolenic acid is present. Taken together, these data suggest that the native TM domain of TcpH enables enhanced association within TI fractions (i.e., lipid-ordered membrane domains), and thereby supports elevated TcpP abundance within the TI fraction, during growth with α-linolenic acid.

Prior studies revealed that studying lipid-ordered membrane domains with this biochemical method can yield dramatically different results with changes in detergent concentration and temperature (90). We thus performed similar experiments using an alternative TI extraction method, which still relies on Triton X-100, that differs in cell lysis temperature. Using this method, we observed the same trend of increased TcpH and TcpP abundance within the TI fraction in WT cells with α-linolenic acid present (Fig. S6). Similarly, we did not detect an increase in TcpP or _EpsM_TcpH abundance in the TI fraction when α-linolenic acid was present for _EpsM_TcpH cells (Fig. S6). Lastly, our data indicate that both arachidonic and docosahexaenoic acid do not stimulate TcpH-dependent protection of TcpP. Thus, to further test our model, we quantified TcpP and TcpH abundance in TI and TS membrane fractions upon exposure to either arachidonic or docosahexaenoic acid (Fig. S7). We did not observe any change in the abundance of TcpH or TcpP within TI or TS membrane fractions. These data indicate that the effect of α-linolenic acid on TcpP and TcpH abundance in TI membranes is indeed specific and not the result of our Triton X-100-based biochemical separation of TI and TS membranes.

Excluding _EpsM_TcpH, it remained unclear if α-linolenic acid supplementation induced a general association of membrane proteins to the TI fraction. To test this, we quantified levels of a 19 KDa non-specific membrane protein in TI and TS fractions with and without α-linolenic acid (Fig. S8A). We observed no change in the abundance of this protein in the TI or TS fractions with α-linolenic acid supplementation (Fig. S8A). These data indicate that α-linolenic acid supplementation does not induce a general association of proteins with the TI fraction. Furthermore, we also observed that with α-linolenic acid supplementation, the TI fraction had a higher association of 16:0 fatty acids and a lower association of 18:3 fatty acids than the TS fraction (Fig. S8B). This is consistent with prior studies indicating that lipid-ordered membrane domains are enriched with saturated fatty acids (62).

### TcpP and TcpH interaction is critical for inhibition of RIP

Our data indicate that increased association of TcpP and TcpH molecules in the TI fraction results in enhanced RIP inhibition. The mechanism underlying this RIP inhibition remains unclear. Lipid-ordered membrane domains function as protein concentrators and thereby promote interaction between membrane-localized proteins (58). We hypothesized that enhanced co-association within the TI fraction increased RIP inhibition due to direct interaction between TcpP and TcpH.

To test direct TcpP-TcpH interaction, we used a co-affinity precipitation approach. We genetically fused a His(6 x)-Hsv or Hsv-His(6 x) tag to the C-terminus and N-terminus, respectively, of TcpP, resulting in *tcpP-His-Hsv* and *Hsv-His-tcpP*. We could then extract TcpP from membrane fractions using NTA-Ni beads and identify TcpH and TcpP in elution fractions with ɑ-TcpH and ɑ-Hsv antibody. Proteins tagged at the amino terminus are described with the tag noted first (e.g., Hsv-His-TcpP), while those tagged at the carboxy terminus are described with the tag noted second (e.g., TcpP-His-Hsv).

First, we tested whether both the N- and C- terminally tagged TcpP function like native TcpP by measuring CtxB production. CtxB production was similar to WT, expressing native TcpP, irrespective of which terminus the tag was placed (Fig. S8C).

**Fig 5:**
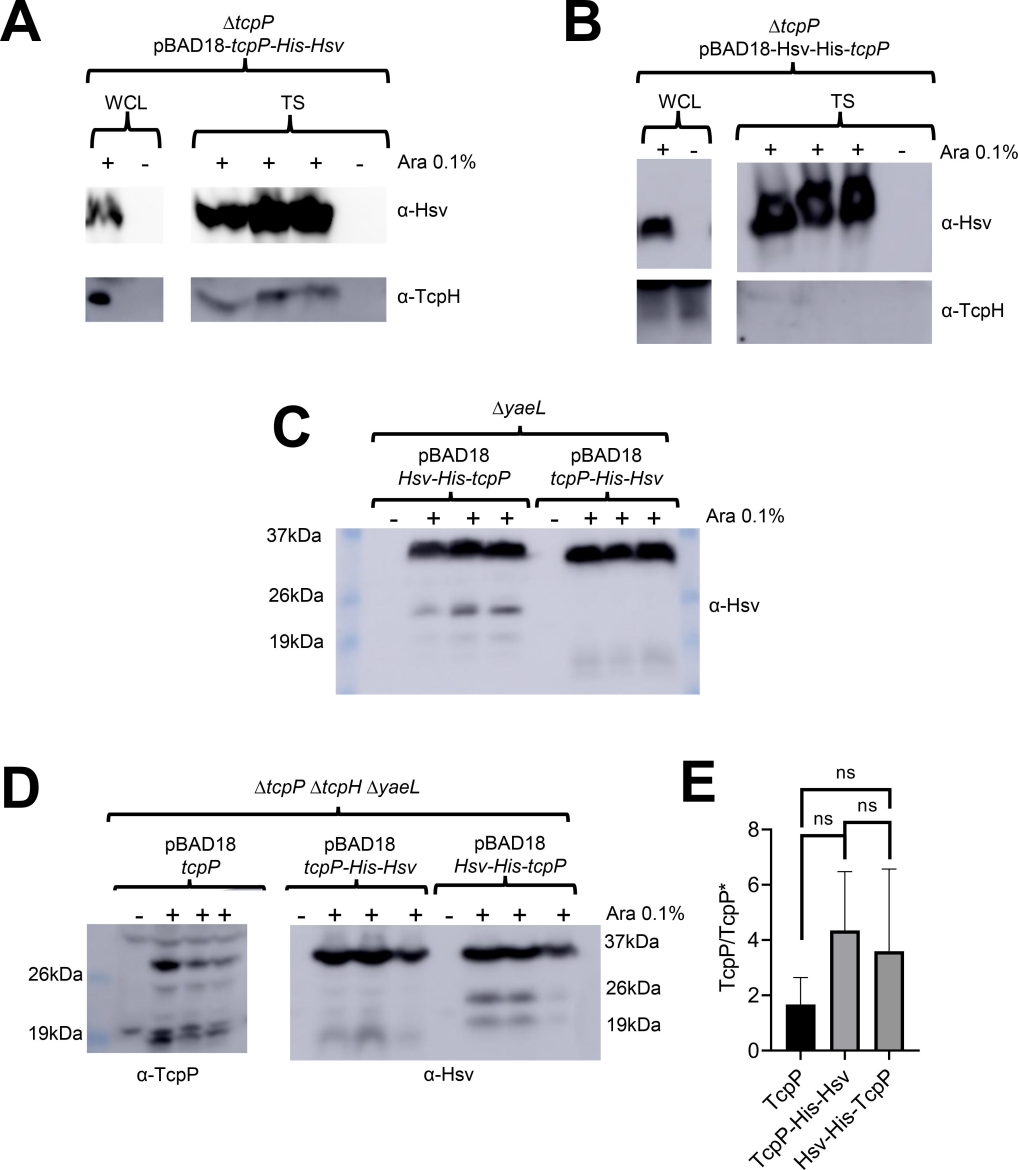
TcpP and TcpH interaction is critical for TcpH-dependent inhibition of RIP. (**A and B**) Co-affinity precipitation of ectopically expressed *tcpP-His-HsV* (**A**), *Hsv-His-tcpP* (**B**). Triton soluble (TS). (**C**) Ectopic expression of *Hsv-His-tcpP* and *tcpP-His-HsV* in Δ*yaeL* cells under virulence-inducing conditions. Hsv-His-TcpP is more sensitive to RIP than TcpP-His-Hsv, as seen by the accumulation of TcpP degradation intermediates between 26 and 19 kDa. (**D**) Ectopic expression of *tcpP* and *tcpP-His-HsV* in Δ*tcpP* Δ*tcpH* Δ*yaeL* cells under virulence inducing conditions. Samples were probed with α-TcpP (left) and α-Hsv (right) antibodies. (**E**) The ratio of full-length TcpP (29 KDa) to other TcpP degradation products (i.e., TcpP*) in western blots found in panel D. Abundance of full-length TcpP and TcpP* was determined *via* densitometry, using ImageJ. ns, indicates no statistical significance. A one-way ANOVA was used to determine statistical significance. (**A–D**) *tcpP* constructs were all ectopically expressed from pBAD18 using arabinose (Ara 0.1% wt/vol). +indicates arabinose was added to the culture. The data here represent samples collected from three independent experiments.

Co-precipitation experiments indicated that the C-terminally tagged TcpP could associate with TcpH, while the N-terminally tagged TcpP could not (Fig. 5A and B). Physical interaction between the C-terminally tagged TcpP and TcpH also correlated to protection from RIP, as determined by lack of accumulation of TcpP degradation intermediates in Δ*yaeL* cells expressing C-terminally tagged TcpP (Fig. 5C). In such cells, the product of Tsp action on TcpP accumulates because the second-site protease YaeL is not present to eliminate it (26, 27). We observed a greater accumulation of TcpP degradation intermediates (between 24 KDa and 19 KDa) in cells expressing N-terminally tagged TcpP compared to those expressing C-terminally tagged TcpP (Fig. 5C). The 24 kDa TcpP degradation intermediate from N-terminally tagged TcpP is also observed in cells expressing native TcpP in the absence of TcpH (Fig. 5C and D). Considering that the N-terminally tagged TcpP is sensitive to RIP even with TcpH present suggests a defect in its association with TcpH and its recognition by the RIP proteases. Despite this defect, N-terminally tagged supports WT CtxB production (Fig. S8C). We conclude this is the result of overexpression of N-terminally tagged TcpP. Native expression of *tcpP* leads to accumulation of only TcpP* in a Δ*tcpH* Δ*yaeL* background (Fig. 1E), but overexpression of *tcpP* in a Δ*tcpP* Δ*tcpH* Δ*yaeL* background yields both full length and TcpP* (Fig. 5D). These data indicate that artificial elevation of TcpP levels, *via* overexpression, can outpace RIP.

Our data demonstrate that TcpP-His-Hsv is less sensitive to RIP in the presence of TcpH. Prior studies have demonstrated that modification of the C-terminus of TcpP can lead to TcpH-independent resistance to RIP (91). To determine whether the addition of His-Hsv to the C-terminus of TcpP promotes resistance to RIP independent of TcpH, we expressed *tcpP-His-Hsv, Hsv-His-tcpP,* and *tcpP* in a Δ*tcpP* Δ*tcpH* Δ*yaeL* background. We observed TcpP degradation intermediates, including TcpP* (~17 KDa), in all strains (Fig. 5D). To determine whether our Hsv-His tagged TcpP variants are equally sensitive to RIP, we quantified the abundance of full-length TcpP and the detected degradation intermediates (Fig. 5E). These data indicate that both N and C-terminally tagged TcpP are equally sensitive to degradation. These data show that the addition of His(6 x)-Hsv to the C-terminus of TcpP does abrogate the need for TcpH to protect TcpP-His-Hsv from RIP (Fig. 5D). In summary, our data indicate that TcpP and TcpH interact and that this interaction is important for inhibition of RIP of TcpP. These data support a model whereby increased association of TcpP and TcpH molecules in the TI leads to enhanced RIP inhibition due to interaction. To further test this hypothesis, we performed additional co-precipitation experiments by expressing TcpP-His-Hsv in cells cultured with and without α-linolenic acid. We found that TcpH co-precipitated with TcpP-His-Hsv in the TI fraction with and without α-linolenic acid (Fig. S9). Due to the poor growth of cells expressing TcpP-His-Hsv in media containing α-linolenic acid, relative to the abundance of TcpP-His-Hsv was lower when α-linolenic acid was present. To account for this growth defect, we normalized TcpH levels to the abundance of TcpP-His-Hsv in the TI fraction. With this normalization, we found that more TcpH co-precipitated when cells were cultured with α-linolenic acid (Fig. S9B). These data support our hypothesis that α-linolenic acid supports elevated interaction between TcpP and TcpH molecules in TI membranes.

It remains unclear why Hsv-His-TcpP is unable to interact with TcpH. Single-molecule tracking studies indicate that TcpP may be sensitive to RIP while interacting with the *toxT* promoter (91). The Hsv tag is enriched with negatively charged amino acids (Hsv amino acid sequence: QP**E**LAP**ED**P**ED**). Given that DNA has an intrinsic negative charge, the addition of Hsv-His(6 x) to the N-terminus of TcpP may promote a conformation similar to the conformation that TcpP molecules adopt when actively interacting with DNA. This hypothesis requires additional experiments to test.

## DISCUSSION

TTRs are broadly distributed and highly diverse among bacteria and archaea (42). Within archaea, TTRs have been found to regulate motility and pilin gene transcription in response to dangerous temperatures and nutrient-limiting conditions (92, 93). Within bacteria, TTRs have been found to regulate bile salt resistance, toxin production, antibiotic resistance, acid resistance, natural competence, pilin/fimbriae transcription, type-3 secretion systems, biofilm formation, metabolism, and have been implicated in the modulation of the human immune system (13, 94–107). In addition, both TcpP and ToxR have accessory proteins, TcpH and ToxS, respectively, that protect them from RIP (14, 25, 26, 48, 108–111). RIP is a form of gene regulation conserved across all domains of life that allows organisms to rapidly respond to extracellular cues, commonly by liberating a transcription factor or a sigma factor, from membrane sequestration (28). Canonical bacterial RIP systems act by releasing an anti-sigma factor fro m the cytoplasmic membrane to influence gene expression (28, 112). TTRs are sensitive to RIP (e.g., CadC) (86). However, RIP of TTRs, such as TcpP, results in their inactivation, typically leading to decreased gene expression. The fundamental mechanisms of RIP for TcpP are understood in terms of the primary proteases that work in the two-step pathway (26, 27), but the regulatory mechanisms influencing these activities have been less well studied. It is clear that TcpH is essential to inhibit RIP of TcpP and that its ability to protect TcpP from RIP changes in response to temperature and pH (14, 25–27). Our data indicate that RIP of TcpP is inhibited by direct interaction with TcpH which regulates the production of virulence factors, CT and TcpA, which thereby impacts the colonization of the gastrointestinal tract. We also provide evidence that α-linolenic acid, a host dietary fatty acid, plays a role in inhibiting RIP by increasing the local concentration of TcpP and TcpH within DRM. This is the first indication that the membrane environment itself influences the activity of both a TTR and a co-component signal transduction system, and these data demonstrate that a dietary fatty acid, in addition to taurocholate, influences TcpP activity (43, 74). Whether α-linolenic acid or other omega-3 or omega-6 fatty acids influences other co-component signal transduction systems remains to be tested.

α-Linolenic acid is an essential omega-3 fatty acid used to synthesize arachidonic and docosahexaenoic acid in humans and mice (113, 114). α-Linolenic acid is acquired *via* dietary supplementation and has health benefits ranging from anti-carcinogenic, anti-atherogenic, anti-inflammatory, improved memory, and anti-diabetic activity (113–130). *V. cholerae* uses exogenous long-chain fatty acids, such as α-linolenic acid, to remodel its phospholipid composition (71, 72). Long-chain fatty acids are transported across the outer membrane by FadL into the periplasmic space where FadD covalently modifies the fatty acids by adding an acyl-CoA group, resulting in the formation of long-chain fatty acyl-CoA (LCFA-CoA) (67–70). Utilization of exogenous fatty acids impacts *Vibrio spp*. pathogenicity, motility, and antibiotic resistance (71, 72, 131). Moreover, *fadL* is highly expression by *V. cholerae* during the colonization of infant rabbits, and loss of *fadL* results in a large fitness disadvantage *in vivo* (132). Our work is aligned with these findings and demonstrates that (i) *toxT* transcription is enhanced in the presence of α-linolenic acid; (ii) TcpP levels are elevated in the presence of α-linolenic acid; (iii) *tcpP* transcription is not increased with exogenous α-linolenic acid; (iv) TcpP and TcpH avidly associate within DRM in the presence of α-linolenic acid; (v) TcpP-TcpH interaction is important for inhibition of RIP; and (vi) enhanced *toxT* expression in the presence of α-linolenic acid is dependent on co-association of TcpP and TcpH in the DRM.

**Fig 6:**
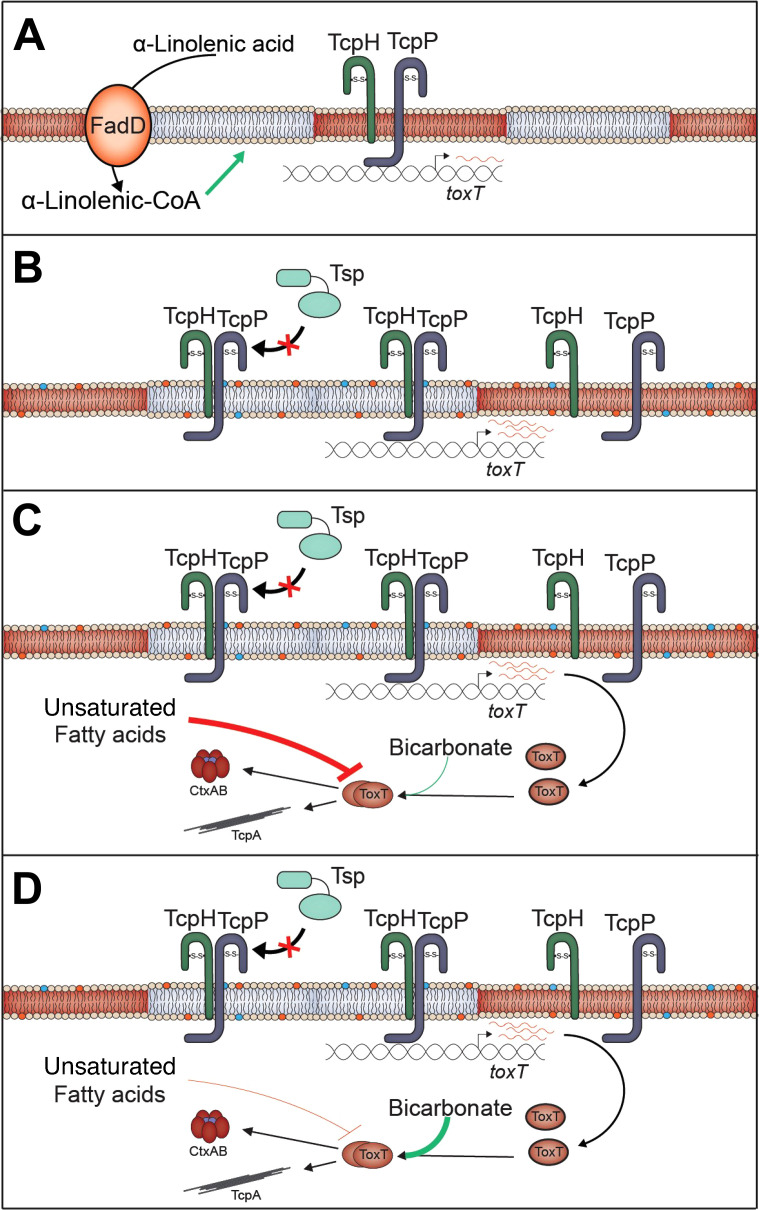
α-Linolenic acid stimulates co-association of TcpP and TcpH within detergent-resistant membranes promoting TcpH-dependent inhibition of RIP. (**A**) Under virulence inducing (Vir Ind) conditions TcpP and TcpH molecules are associated with Triton insoluble (gray; TI) and Triton soluble (red; TS) membrane domains. (**B**) In the presence of exogenous α-linolenic acid, *V. cholerae* cells uptake α-linolenic acid (*via* VolA, FadL) and utilize it directly for phospholipid remodeling *via* the addition of coenzyme A (CoA), *via* FadD (67–70). This leads to changes in the overall phospholipid profile of *V. cholerae,* indicated by the blue and orange phospholipids (68–71). Under these conditions, a majority of TcpP and TcpH molecules transition to the TI membranes leading to enhanced inhibition of RIP by TcpH. The net result of α-linolenic acid supplementation is an increase in *toxT* transcription, indicated by an increase in red *toxT* mRNA. (**C**) In the lumen of the gastrointestinal tract, ToxT activity is thought to be inhibited by unsaturated fatty acids and thus inhibits ToxT-dependent virulence factor expression (75, 76, 131, 133–137). (**D**) Near the surface of epithelial cells, bicarbonate is actively secreted from intestinal epithelial cells and, as such, the concentration of bicarbonate is elevated, including in the crypt of intestinal villi (133–137). As bicarbonate can stimulate ToxT activity, ToxT-dependent virulence gene expression is proposed to be stimulated near epithelial cells (133–137).

Our data support a model where, once present in the gastrointestinal tract (GI), *V. cholerae* cells take up and incorporate α-linolenic acid, present in the GI tract of infant mice (Fig. 2D), into phospholipids, thereby altering the composition of the cytoplasmic membrane. This influences TcpH and TcpP molecules to increase their association with lipid-ordered membrane domains. n-3 polyunsaturated lipids (i.e., omega-3 fatty acids) are known to increase lipid-ordered domain size in eukaryotes by promoting aggregation of existing lipid-ordered membrane microdomains (83, 84). As lipid-ordered membrane domains are known to be relatively small in size (6–200 nm) (58, 60), we hypothesize that this leads to an increase in the local concentration of TcpP and TcpH molecules, thereby allowing TcpH to enhance RIP inhibition of TcpP *via* increased interactions with TcpP (Fig. 6).

Previous studies have investigated the role of exogenous fatty acids in the pathogenesis of *V. cholerae*. These concluded that FadD is required for wild-type *toxT* expression through a mechanism involving its effect on TcpP levels (138, 139). These prior publications support our model as an accumulation of α-linolenic acid in the periplasmic space or within the cytoplasmic membrane, due to loss of *fadD,* results in a reduction in TcpP levels, rather than an increase (138, 139). This work indicates that free α-linolenic acid (i.e., not incorporated in phospholipids) within the periplasmic space, cytoplasm, or within the cytoplasmic membrane, does not promote TcpH-mediated inhibition of RIP. In conjunction with the data presented here, this indicates that α-linolenic acid needs to be incorporated into the cytoplasmic membrane as a phospholipid to influence TcpH function.

Transmembrane domain length and surface area are major factors in determining the preference of a protein for lipid-ordered (enriched with proteins having longer TM domain and low surface area) or lipid-disordered (enriched with proteins having shorter TM domain and high surface area) membrane domains (140). We demonstrated that TcpH and TcpP increase localization within DRM domains in the presence of α-linolenic acid while _EpsM_TcpH does not (Fig. 4). _EpsM_TcpH has a shorter TM domain than TcpH (20 amino acids vs 22 amino acids) and a higher overall surface area (108 Å^2^ vs 92 Å^2^). Our data suggests that the TM domain properties of _EpsM_TcpH molecules inhibit its transition from the TS fraction to the TI fraction in the presence of α-linolenic acid, and thereby the ratio of _EpsM_TcpH to TcpP molecules within the TI fraction is insufficient to enhance the protection of TcpP and support elevated *toxT* expression. In support of this, the TM domain of TcpH is also highly conserved across *V. cholerae* strains (Fig. S3). Alternatively, it is also possible that TcpH, and not _EpsM_TcpH, undergoes post-translational modification (e.g., palmitoylation) within its TM domain. We view this as unlikely as TcpH is not predicted to have a palmitoylation site within its TM domain. Furthermore, TcpH-like proteins in other co-component signal transduction systems in *Salmonella enterica* serovar Typhimurium and *Yersinia pseudotuberculosis* also have comparable surface area per TM residue to TcpH (STM0345: 97 Å^2^, BZ17_3282: 94 Å^2^, BZ17_3565: 101Å^2^). This indicates that host dietary fatty acids may also influence these co-component systems in *S*. Typhimurium and *Y. pseudotuberculosis*.

Unexpectedly, _EpsM_TcpH supports an overall higher level of *toxT* gene expression than wild-type TcpH, despite having lower levels of TcpP with α-linolenic acid present. It is unclear how this is occurring. Super-resolution imaging experiments aimed at understanding how TcpP molecules locate the *toxT* promoter from the cytoplasmic membrane revealed that TcpP molecules transition to a slow diffusion state before interacting with the *toxT* promoter (91, 141, 142). As EpsM has been shown to interact with EpsL (a component of the Eps Type-II secretion system) (65), we hypothesize that elevated levels of *toxT* expression in _EpsM_TcpH are due to reduced TcpP diffusion rates via interaction with the Type-II secretion system (i.e., TcpP-epsMTcpH-Eps).

In addition, it also appears that the surface area of the transmembrane domain of TcpP influences its function. Prior analysis of TcpP transmembrane domain revealed that mutation of L152 and W162/S163 with alanine (which reduces the overall surface area of the transmembrane domain) increased *toxT* expression (143). It remains unclear why these mutations increase TcpP function, but given the data presented here, it is possible that TcpPL152A and TcpP W162A/S163A may have a greater propensity than TcpP, in the absence of α-linolenic acid, to associate within DRMs (i.e., lipid-ordered membrane domain). Prior work has also noted that many TTRs have a similar transmembrane surface area and also an overall lower amount of sensory domains (42). Further indicating that TTRs, like TcpP, respond to the cytoplasmic membrane.

Based on our data here and other literature, we hypothesize that phospholipid remodeling of *V. cholerae* occurs in the lumen during the initial stages of infection. Our data suggest that this remodeling promotes TcpH-mediated inhibition of RIP and promotes *toxT* transcription. However, unsaturated fatty acids can also inhibit the degradation and activity of ToxT (73, 74, 133). This likely prevents premature expression of TCP which is known to stimulate microcolony formation and thereby could inhibit penetration of the mucus layer (134). Bicarbonate present at high concentrations at the surface of epithelial cells, competes with unsaturated fatty acids to activate ToxT once *V. cholerae* reaches the surface of epithelial cells, its primary site of infection (135–137). There is also evidence that bicarbonate represses *toxT* transcription (136). This indicates that expression of *toxT*, stimulated by enhanced RIP antagonism, during early infection (i.e., the lumen) is critical for *V. cholerae* to cause disease. Due to the essential nature of omega-3 and omega-6 fatty acids, it is technically challenging to test the role of α-linolenic acid directly *in vivo* and will require future sophisticated *in vivo* studies. Evaluating the *in vivo* model proposed here will be the subject of future experiments. α-Linolenic acid represents the first *in vivo* signal that modulates RIP of TcpP, and, to the best of our knowledge, the first evidence that lipid-ordered and lipid-disordered membrane domains exist in *V. cholerae*. The data presented here further expand our knowledge of the complex virulence regulatory cascade in *V. cholerae,* our knowledge of rapidly evolving co-component signal transduction systems in *enterobacteria,* and TTRs in bacteria and archaea.

## MATERIALS AND METHODS

### Bacterial culture conditions

Unless otherwise stated *Escherichia coli* and *V. cholerae* were grown at 37°C in Luria-Bertani broth (LB) with vigorous shaking (210 rpm). LB was prepared as previously described (144). To stimulate virulence factor production, *V. cholerae* strains were subcultured, to an O.D. of 0.01, and grown under virulence inducing conditions (Vir Ind; 30°C, LB pH 6.5 ± 0.5, and 110 rpm) or non-virulence inducing conditions (non-Vir Ind; 37°C, LB pH 8.5 ± 0.5, and 210 rpm). Media used for both Vir Ind and non-Vir Ind were sterilized using 1L 0.22 µm vacuum filtration units (Sigma) after pH adjustment. Unless otherwise stated, antibiotics were used at the following concentrations: ampicillin (100 µg/mL), chloramphenicol (30 µg/mL), streptomycin (100 µg/mL), and cerulenin (10 µg/mL). Overexpression of constructs by pBAD18 was induced by culturing strains in LB containing 0.1% arabinose. For additional information on growth conditions for *ex-vivo* mouse fecal experiments, experiments with components of crude bile (including α-linolenic acid), and a complete list of bacterial strains see SI Appendix, Materials and Methods and Table S2.

### Plasmid construction

Briefly, DNA fragments 500 bp upstream and downstream of the target gene were connected to the DNA insert by splicing *via* overlap extension PCR (Thermo Scientific) (see Table S3). Plasmid vectors were then digested with KpnI-HiFi and XbaI (New England BioLabs) at 37°C for 2 hours. Insert and vector fragments were then added to the Gibson assembly master mix (New England BioLabs) and incubated at 50°C for 30 minutes. Plasmids were then introduced to *E. coli* ET12567 *∆dapA* (λpir +) by electroporation. pKAS32 plasmids were then transferred to *V. cholerae* strains *via* mating on LB agar plates at 30°C overnight. pBAD18 plasmids were introduced into *V. cholerae* strains *via* electroporation.

### Mutant construction

Briefly, mutants were constructed using allelic exchange as previously described (145). *V. cholerae* harboring pKAS32 derivatives were grown in 2 mL LB for 2 hours (37°C), and then an additional 2 hours with added streptomycin (2,500 µg/mL). After a total of 4 hours of incubation, 20 µL of culture was spread on LB agar plates containing streptomycin (2,500 µg/mL) and incubated at 37°C overnight. Colonies that were resistant to streptomycin were screened *via* colony PCR. Mutants were confirmed by sequencing the region of interest (GeneWiz).

### Growth curves

*V. cholerae* strains were subcultured from an overnight culture to a final optical density (600 nm) of 0.01 in 200 µL of virulence-inducing media, LB, or M9 minimal media (supplemented with 0.05% glucose) per well of a 96-well plate. The plate was then incubated at 30°C or 37°C in a SPECTROstar Omega plate reader (BMG LABTECH), with shaking and optical density measurements every 30 minutes.

### Western blots

Western blots were performed as previously described (26). Briefly, after cell lysis, samples were normalized by total protein concentration, determined *via* a Bradford assay or Bicinchoninic acid assay (Sigma Aldrich). Samples were run on SDS page gels (12.5% acrylamide) for 1.5 hours at 90–120 volts and then transferred to nitrocellulose membranes overnight at 35 mA or for 2 hours at 200 mA. Membranes were blocked with 5% non-fat milk, 2% bovine serum albumin, 0.5% Tween-20, in Tris-buffered saline for 1 hour followed by incubation with primary antibodies for 1 hour. Membranes were washed three times with Tris-buffered saline. Secondary antibodies were incubated with the membrane for 1 hour. Membranes were washed three times with Tris-buffered saline and then incubated with SuperSignal HRP Chemiluminescence substrate (Thermo Fisher). Membranes were imaged with an Amersham Imager 600. For additional information see SI Appendix, Materials and Methods.

### Enzyme-linked-immunosorbent assay

Enzyme-linked immunosorbent assay (ELISAs) were performed as previously described (26, 146). Briefly, 10 µL of culture supernatant was added to 140 µL PBS-T (phosphate-buffered saline, 0.05% Tween-20, 0.1% BSA) in row A of plates coated with GM1 (monosialotetrahexosylganglioside) and then diluted down each column. After 1 hour of incubation, plates were then washed with PBS-T three times. Primary (α-CtxB 1:8000, Sigma Aldrich) and secondary antibodies (Goat anti-Rabbit IgG-HRP 1:5,000, Sigma Aldrich) were diluted in PBS-T. 100 µL of diluted antibody was added to each well and incubated for 1 hour at room temperature, with three rounds of washing after incubation. 100 µL of TMB (3,3′,5,5′-tetramentylbenzidine, Sigma) was added to each well and the reaction stopped by addition of 100 µL of 2M sulfuric acid. The optical density (450 nm) was measured for each well using SPECTROstar Omega plate reader (BMG LABTECH). For additional information, see SI Appendix, Materials and Methods.

### Infant mouse colonization

Infant mouse colonization experiments were performed as previously described (147, 148). Briefly, 3- to 6-day-old infant mice were orogastrically inoculated with ~1×10^6^ bacterial cells. Infant mice were kept at 30°C in sterile bedding and euthanized either 18 hours or 21 hours after infection. For fluid accumulation studies, a higher inoculum dose was used (~1×10^8^) and infant mice were weighed prior to collection of mouse intestines, and mouse intestines were weighed after blotting on absorbent paper. Homogenates were then serially diluted in PBS, spread on LB plates containing streptomycin, and incubated at 37°C overnight. For additional information see SI Appendix, Materials and Methods.

### Real-time quantitative PCR (RT-qPCR)

RT-qPCR experiments were performed as previously described (149). Briefly, RNA was preserved in 1 mL of Trizol (Sigma Aldrich) and then extracted from cells using an RNEasy kit (Qiagen). RNA was then treated with Turbo DNase. cDNA was generated from DNase-treated RNA using Superscript III reverse transcriptase (Thermo Scientific) as previously described (150). 5 ng of cDNA was used with SYBR green master mix (Applied Biosystems) to perform the RT-qPCR. *recA* was used as a housekeeping gene (150). See Table S3 for primers. For additional information, see SI Appendix, Materials and Methods.

### β-Galactosidase activity assay

β-galactosidase activity and Miller units were determined as previously described (151). Cells were resuspended in 1 mL of Z-buffer (Na_2_HPO_4_ 60 mM, NaH_2_PO_4_ 40 mM, KCl 10 mM, MgSO_4_ 1 mM, β-mercaptoethanol 50 mM, pH 7.0). Cells were permeabilized with 60 µL of SDS (0.1%) and chloroform and then incubated at 30°C for 10 minutes. 200 µL of ortho-Nitrophenyl-ß-galactoside (4 mg/mL) was added and incubated at room temperature until a color change was observed. 500 µL of sodium bicarbonate was added to stop the reaction. The optical density for each sample was measured (at both 420 nm and 55 nm). For additional information, see SI Appendix, Materials and Methods.

### Triton X-100 subcellular fractionation

Cells were collected and washed with PBS. For spheroplast fractionation, cells were resuspended in 100 µL of 200 mM Tris HCl. After resuspension, components were added sequentially to each sample: 200 µL of 200 mM Tris HCl and 1M sucrose, 20 µL of 10 mM EDTA, 20 µL of lysozyme (10 mg/mL), 10 µL of protease inhibitor cocktail (Sigma), and 600 µL of H_2_O. Samples were then incubated at room temperature for 30 minutes. After room temperature incubation, 700 µL of 2% Triton X-100, 50 mM Tris HCl, and 10 mM MgCl_2_ were added. For gentle cell lysis, pelleted cells were resuspended in 10 mL of TS buffer (1% Triton X-100, 10 mM imidazole, 500 mM HEPES, 10% glycerol, 2M MgCl_2_). Samples then underwent three rounds of freeze-thaw lysis in 180-proof ethanol at −80°C. Triton X-100 soluble and insoluble membrane fractions were then separated by ultracentrifugation (100,000 × *g* 1 hour). The supernatant (i.e., the Triton X-100 soluble fraction; TS) and the pellet (i.e., the Triton X-100 insoluble fraction; TI) were collected. The TI fraction was resuspended in 500 µL of TI buffer (1% Tween 20, 0.5M MOPS, and 10 mM imidazole). The TS fraction was concentrated using Amicon protein concentrators with a 10 KDa cutoff (Sigma).

### Subcellular fractionation

Cells were fractionated following the Tris-sucrose-EDTA method (152, 153). Briefly, spheroplast fractions were resuspended in 500 µL 0.45% NaCl. To lyse the spheroplasts, 50 µL of 10% SDS was added, and samples were then boiled for 5–10 minutes. Periplasmic fractions were concentrated using trichloroacetic acid (TCA) (1, 2). Pelleted whole cells were resuspended in 50–200 µL of resuspension buffer (50 mM Tris-HCl, 50 mM EDTA, pH 8.0). Cells were then lysed by the addition of lysis buffer (10 mM Tris-HCl, 1% SDS) and boiled for 5–10 minutes. All fractions were stored at −20°C until use. For additional information see SI Appendix, Materials and Methods.

### Co-affinity precipitation

*V. cholerae* strains were grown under Vir Ind for 6–8 hours. After incubation, cells were resuspended in PBS, proteins were cross linked by the addition of 1 mM Dithiobis (succinimidyl propionate) or 1 mM Suberic acid bis (N-hydroxysuccinimide ester) and incubated on ice for 30 minutes. 50 µL of Tris HCl pH 8.5 was added (1M final concentration) and samples were incubated on ice for an additional 15 minutes. Cells were then pelleted (2,450 × *g* 15 minutes) and TI and TS fractions were collected *via* the gentle cell lysis method. After collection of TI and TS fractions, 100 µL of washed His-affinity gel (i.e., Ni-NTA Magnetic Agarose Beads) (ZYMO Research) and 10 µL of protease inhibitor cocktail (Sigma) were added to the TI and TS fractions, and samples were incubated on a rocking platform overnight at 4°C. Samples were then centrifuged to collect the Ni-NTA agarose beads (2,450 × *g* 15 minutes). After collection, the Ni-NTA agarose beads were washed three times with their respective buffers (i.e., TS buffer for TS samples). An equal volume of laemmli buffer was added to each sample (BIO-RAD) and then boiled for 5 minutes. Boiled samples were then used directly for western blot analysis.

### Fatty acyl methylester analysis

Analysis of fatty acids from whole *V. cholerae* cells was done as previously described (154). Briefly, cells were lysed *via* the addition of 300 µL of extraction solvent [composed of methanol, chloroform, and formic acid (20:10:1, vol/vol/vol)]. After lipids were extracted, the fatty acyl methylester (FAME) reactions were carried out as described (154). After the FAME reactions, fatty acid content was measured *via* Gas-Liquid Chromatography using a DB-23 column (Agilent, part number: 122–2332). Molar values of each peak were then normalized to an internal standard (15:0) to calculate the total molar percentage of each fatty acid detected.

### Membrane fluidity

Membrane fluidity was measured as previously described (155) using a membrane fluidity kit that quantifies the fluorescence of a lipophilic dye (Pyrenedecanoic acid) (Abcam). Ethanol was used as a positive control (156). Briefly, WT and _EpsM_TcpH cells were grown under specified conditions for 8 hours, for additional information see SI Appendix, Materials and Methods. After incubation, cells were collected from 1 mL of culture and resuspended in 500 µL LB. Cells were incubated with the fluorescent lipid reagent for 30 minutes at room temperature. Cells were then washed twice with LB and fluorescence (excitation, 350 nm, and emission, 400 nm and 470 nm) was quantified for each sample. Unlabeled cells and non-Vir ind conditions were used as negative controls.

### Mass spectrometry

Sample fatty acid and metabolite extraction was performed by protein precipitation with ethanol, as previously described (157). For each sample, 20 ng of d8-arachidonic acid was used as an internal control. Samples were homogenized in a bead mill for 2 minutes (ThermoFisher). Samples were incubated at –20°C for 1 hour and then protein was removed by centrifugation (15,000 x *g* for 20 minutes). The supernatants underwent an additional round of protein precipitation, as described above. Samples were dried *via* speedvac, and then reconstituted in 200 µl of acetonitrile. Chromatographic alignment, isotope correction, peak identification, and peak area calculations were performed using MAVEN software. Concentrations of each analyte were determined against the peak area of the d8-arachidonic acid internal standard. Additional fatty acids (C24–C28) were identified by comparison against certified reference materials and natural products as no reference standards are readily available. For additional information, see SI Appendix, Materials and Methods

## SUPPLEMENTAL MATERIAL

Supplemental material mbio.00721-24-s0001.docx Supplemental methods, tables, and figure legends.

Figure S1 mbio.00721-24-s0002.tif Growth rates of transmembrane and periplasmic TcpH variants are similar to WT cells.

Figures S2 and S3 mbio.00721-24-s0003.tiff Constructs in adult mice and conservation of TM domain.

Figure S4 mbio.00721-24-s0004.tif α-Linolenic acid stimulates *toxT* transcription in a TcpH-dependent manner.

Figure S5 mbio.00721-24-s0005.tif α-Linolenic acid is utilized by *V. cholerae* cells, increases membrane fluidity, and does not influence *tcpP* transcription or promote non-specific protein association within detergent-resistant membranes.

Figure S6 mbio.00721-24-s0006.tif Spheroplast TI/TS membrane extraction demonstrates that TcpP and TcpH increase in abundance in TI membrane fractions with α-linolenic acid.

Figure S7 mbio.00721-24-s0007.tif Arachidonic and docosahexaenoic acid do not change the abundance of TcpP or TcpH in TI and TS membrane fractions.

Figure S8 mbio.00721-24-s0008.tif α-Linolenic acid does not promote non-specific protein association within detergent-resistant membranes, and Hsv-His(6×)-tagged TcpP constructs remain functional.

Figure S9 mbio.00721-24-s0009.tif α-Linolenic acid promotes interaction between TcpP and TcpH within the TI membrane fraction.

Figure S10 mbio.00721-24-s0010.tif Western blots used for densitometry analysis.

ASM does not own the copyrights to Supplemental Material that may be linked to, or accessed through, an article. The authors have granted ASM a non-exclusive, world-wide license to publish the Supplemental Material files. Please contact the corresponding author directly for reuse.
