# Supplementary material for: An essential host dietary fatty acid promotes TcpH inhibition of TcpP proteolysis promoting virulence gene expression in Vibrio cholerae
Source: mBio. 2024 Jul 3;15(8):e00721-24. doi: 10.1128/mbio.00721-24 (PMC11323476; doi:10.1128/mbio.00721-24)
Supplement: Supplemental material — Supplemental methods, tables, and figure legends. [file mbio.00721-24-s0001.docx]

**Supplementary Information for**

**An** **essential host dietary fatty acid promotes TcpH dependent inhibition of regulated intramembrane proteolysis of TcpP in *Vibrio cholerae***

Lucas M. Demey^1^, Ritam Sinha^1^, and Victor J. DiRita^1*^

^1^Department of Microbiology & Molecular Genetics, Michigan State University, East Lansing, Michigan, 48824; USA.

*Address correspondence to Victor J. DiRita

**Email:** [diritavi@msu.edu](about:blank)

**SI Materials and Methods**

**Bacterial culture conditions**

*ex-vivo* mouse fecal experiments with sterile and non-sterile mouse fecal media were conducted aerobically at 37°C in 48 well plates (Sigma) with shaking (210 rpms). Sterile mice fecal samples were collected from C57 Black female mice on 4 separate days and stored at -80°C. After collection mice fecal samples were homogenized, via mortar and pestle, and then suspended in M9 minimal media. The final concentration of mice fecal media was 9% w/v. The mice fecal media was then spun down (2450xg for 10 min) to remove insoluble material. The supernatant was collected, and filter sterilized using a 0.45 µM syringe filter (Sigma). Non-sterile mice fecal samples were collected from C57 Black female mice on three separate days. Mice fecal matter was directly resuspended in M9 media to a final concentration of 9% w/v. Mice fecal media was then incubated at room temperature for 1 hour while shaking on a tabletop shaker. Mice fecal media was spun down (2450xg for 10 min). The supernatant was collected and used directly for the growth curve. *V. cholerae* cell density was determined by counting CFU’s on LB agar plates supplemented with streptomycin. Microbiota in mice fecal matter were not found to be resistant to streptomycin.

To test if crude bile (Ox gal, Sigma Aldrich), as well as components of crude bile, we opted to pretreat all *V. cholerae* strains under Vir Ind conditions before exposing cells to these additional factors. Briefly, *V. cholerae* cells were subcultured from overnight cultures to an optical density of 0.01 in 100 ml of LB pH 6.5 in a 250 ml Erlenmeyer flask. *V. cholerae* strains were grown for 4 hours under Vir Ind conditions, centrifuged (2450 X g 15 minutes), resuspended in 0.8 ml LB. 200 µl of resuspended cells were transferred to 50 ml of fresh Vir Ind media in 125 ml erlenmeyer flasks. The following were supplemented to Vir Ind media: crude bile (CB; final concentration 0.4% v/v), α-linolenic acid (LA), linoleic acid (LI), palmitic acid (PA), stearic acid (SA) , docosahexaenoic acid (DH), arachidonic acid (AC), purified bile salts cholate and deoxycholate (PB; final concentration 100µM). All compounds were purchased from Sigma Aldrich. CB and PB were solubilized in Vir Ind media and filter sterilized (0.22 µM; Sigma). LA, LI, SA, PA, DH, and AC were dissolved in Dimethyl sulfoxide (DMSO) and then added to Vir Ind media. LA, LI, SA, PA, DH, and AC sterility were confirmed by spreading 100µl of DMSO solubilized fatty acid on LB agar plates (data not shown). Unless otherwise states, the final concentration of LA, LI, SA, PA, DH, and AC was 500µM.

**Mutant construction**

*V. cholerae* harboring pKAS32 derivatives were grown in 2 ml LB for 2 hours (37ºC), and then an additional 2 hours with added streptomycin (2500 µg/ml). After a total of 4 hours of incubation, 20 µl of culture was spread on LB agar plates containing streptomycin (2500 µg/ml) and incubated at 37ºC overnight. Colonies that were resistant to streptomycin were screened via colony PCR. Mutants were confirmed by sequencing the region of interest (GeneWiz).

**Western blots**

After cell lysis, the total protein concentration of each sample was measured via Bradford assay (Sigma Aldrich). Samples were subsequently diluted to a final concentration of 0.5 µg total protein/µl. All SDS page gels contained 12.5% acrylamide and were run at 90-120 volts for 1.5 hours. Proteins were transferred to nitrocellulose membranes using a semi dry electroblotter (Fisher Scientific) overnight at 35 mA or for 2 hours at 200mA. Membranes were blocked with 15 ml of blocking buffer (5% non-fat milk, 2% bovine serum albumin, 0.5% Tween-20, in Tris-buffered saline) for 1 hour at room temperature. Primary antibodies were diluted in 5% non-fat milk and Tris-buffered saline (α-TcpH 1:500, α-TcpP 1:1,000, and α-TcpA 1:100,000) and incubated with the membranes for 1 hour at room temperature. Membranes were washed three times for 5-15 minutes with Tris-buffered saline. Secondary antibodies (Sigma Aldrich) were diluted in 5% non-fat milk in Tris-buffered saline (Goat anti-Rabbit IgG-HRP 1:2,000) and incubated as before. Membranes were washed three times for 5-15 minutes with Tris-buffered saline and then incubated with SuperSignal HRP Chemiluminescence substrate (Thermo Fisher). Membranes were imaged with an Amersham Imager 600.

**Enzyme Linked-Immunosorbent Assay (ELISA)**

*V. cholerae* cells were subcultured from overnight cultures to an optical density of 0.01 in 10 ml of LB pH 6.5. Cultures were incubated at 30ºC for a total of 24 hours. Cells were collected by centrifugation at 2450X g for 15 minutes. 1 ml of culture supernatant was collected and the remaining supernatant was discarded. All steps of EILSA were performed at room temperature. 10 µl of culture supernatant was added to 140 µl PBS-T (phosphate buffered saline, 0.05% Tween-20, 0.1% BSA) in row A of plates coated with GM1 (monosialotetrahexosylganglioside). Samples were diluted (1:3) down each column and incubated at room temperature for 1 hour. Plates were then washed with PBS-T three times. Primary (α-CtxB 1:8000, Sigma Aldrich) and secondary antibody (Goat anti-Rabbit IgG-HRP 1:5,000, Sigma Aldrich) were diluted in PBS-T. 100 µl of diluted antibody was added to each well and incubated for 1 hour at room temperature. Plates were again washed with PBS-T as before. 100 ul of TMB (3,3',5,5'-tetramentylbenzidine, Sigma) was added to each well and incubated for 5-10 minutes. The reaction stopped by addition of 100 µl of 2M sulfuric acid and the optical density (450 nm) was measured for each well using SPECTROstar Omega plate reader (BMG LABTECH).

**Infant Mouse Colonization**

The Institutional Animal Care and Use Committee at Michigan State University approved all animal experiments (PROTO201900421). 3-6 day old male and female Infant mice (CD1, catalog #: 022CD1) were purchased from Charles River (Wilmington, MA). Infant mice were transported with a female adult mouse. Upon arrival, infant mice were separated from the adult female mouse, 2 hours prior to infection, and all adult female mice were euthanized. Infant mice were randomly assigned into infection groups. 70% ethanol was used to sterilize gloves, tubing, and surfaces between groups. The number of mice used per group is indicated in the figure or figure legend. Briefly, three- to six- day old CD-1 mice were orogastrically inoculated with ~1x10^6^ or ~1x10^8^ bacterial cells after 2 hours of separation from their mothers. Infant mice were kept at 30ºC in sterile bedding and euthanized either 18 hours or 21 hours after infection. Mouse intestines (small and large) were weighed in 3 ml PBS and homogenized. For fluid accumulation studies, infant mice were weighed prior to collection of mouse intestine, and mouse intestines were weighed after blotting on absorbent paper. Homogenates were then serially diluted in PBS, spread on LB plates containing streptomycin, and incubated at 37ºC overnight.

**Real-time quantitative PCR (RT-qPCR)**

RNA was preserved by resuspending *V. cholerae* cells in 1 ml of Trizol (Sigma Aldrich) and then extracted from cells using an RNEasy kit (Qiagen) according to manufacturer’s instructions. RNA was then treated with Turbo DNase for 30 minutes at 37ºC. After DNase treatment, RNA quality was determined by detection of large and small ribosomal subunits via 2% agarose gel. RNA quantity was then measured using a Nanodrop spectrophotometer (Thermo Scientific). cDNA was generated from DNase treated RNA using Superscript III reverse transcriptase (Thermo Scientific) as previously described  [(1)](about:blank). 5 ng of cDNA was used with SYBR green master mix (Applied Biosystems) to perform the RT-qPCR. *recA* was used as a housekeeping gene of reference to calculate the threshold values (ΔΔC_T_). See Table S3 for primers.

**β-Galactosidase activity assay**

*V. cholerae* cells were subcultured from overnight cultures to an optical density of 0.01 in 50 ml of LB pH 6.5. *V. cholerae* strains were grown for 4 hours under Vir Ind conditions. Following incubation cultures were centrifuged (2450 X g 15 minutes), resuspended in 1 ml LB, and then 200 µl of the culture resuspension was transferred to fresh media (Vir Ind, Vir Ind supplemented with crude bile/ cholate and deoxycholate (purified bile)/ α-linolenic acid, or non-Vir Ind). Cultures were grown for an additional 4 hours under their indicated condition. At the indicated time point (4 hours or 8 hours) 1.5 ml of culture was removed, centrifuged (4000 X g 15 minutes), and resuspended in 1 ml of Z-buffer (Na_2_HPO_4_ 60mM, NaH_2_PO_4_ 40mM, KCl 10mM, MgSO_4_ 1mM, β-mercaptoethanol 50mM, pH7.0). Cells were permeabilized with 60 µl of SDS (0.1%) and chloroform and incubated at 30ºC for 10 minutes. 200 µl of ortho-Nitrophenyl-ß-galactoside (4mg/ml) was added to lysed cells and incubated at room temperature until a color change was observed. 500 µl of sodium bicarbonate was added to stop the assay. The optical density for each sample was measured (at both 420nm and 55nm). β-galactosidase activity is displayed as Miller units. Miller units = $\frac{{OD}_{420}-(1.75*{OD}_{550})}{{OD}_{600}*minutes*ml}*1000$.

**Subcellular Fractionation**

*V. cholerae* cells were subcultured from overnight cultures to an optical density of 0.01 in 50 ml of LB pH 6.5. After 2 hours of incubation, plasmids were induced by the addition of arabinose (final concentration of 0.1%) at 30ºC with mild shaking (110 rpm), and then cultured for an additional 5 hours. All steps of the fractionation procedure were performed on ice. Spheroplast fractions (i.e., cytoplasm and the cytoplasmic membrane) were resuspended in 500 µl 0.45% NaCl. To lyse the spheroplasts 50 µl of 10% SDS were added and samples were then boiled for 5-10 minutes. Periplasmic fractions were concentrated using trichloroacetic acid (TCA) [(2, 3)](about:blank). Pelleted whole cells were resuspended in 50-200 µl of resuspension buffer (50mM Tris-HCl, 50mM EDTA, pH 8.0). Cells were then lysed by the addition of lysis buffer (10mM Tris-HCl, 1% SDS) and boiled for 5-10 minutes. All fractions were stored at -20 ºC until use.

Soluble and insoluble fractionation of *V. cholerae* cells was performed as described by Miller *et. al.*, with modifications [(4)](about:blank). Initial steps of the Tris-sucrose-EDTA extraction were followed regarding growth and collection of *V. cholerae* cells. Following collection, cells were resuspended in 10 ml of lysis buffer (10 mM Tris HCl pH 8.0, 750mM sucrose, EDTA-free protease inhibitor, 2mM EDTA, 50 µg/ml lysozyme, 10 U/ml DNase 1) and incubated on ice for 20 minutes. Cells underwent two rounds of lysis via French press (7,000-10,000 psi). Cellular debris was removed by centrifugation (1200 X g for 10 minutes) and supernatant was retained. Insoluble (i.e., the inner and outer membrane) and soluble fractions were separated by ultracentrifugation (100,000 x g for 2 hours at 4ºC). The pellet, containing the membrane fraction, was collected and resuspended in 500 µl 5mM EDTA and 25% sucrose. The insoluble membrane fraction underwent a second round of ultracentrifugation and was then collected. All samples were stored at -80ºC until further use.

**Fatty Acyl Methylester (FAME) analysis**

Briefly, *V. cholerae* cells were grown with and without linolenic acid (500 µM) as described in the section below. Cells were collected by centrifugation (2450 X g 15 minutes) and then washed with PBS. Cells were then lysed via addition of 300 μl of extraction solvent (composed of methanol, chloroform and formic acid [20:10:1, v/v/v]). After lipids were extracted the Fatty Acyl Methylester (FAME) reactions were carried out as described [(5)](about:blank). After the FAME reactions, fatty acid content was measured via Gas-Liquid Chromatography using a DB-23 column (agilent, part number: 122-2332). Molar values of each peak were then normalized to an internal standard (15:0) to calculate the total molar percentage of each fatty acid detected.

**Membrane Fluidity**

Membrane fluidity was measured using a membrane fluidity kit (Abcam). Pyrene decanoic acid exists in monomeric and dimeric states within membranes. Dimerization of pyrene decanoic acid occurs in areas of low fluidity (or low viscosity) and results in a change in its emission spectra. Thus, the ratio of dimeric (excimer state) and monomeric pyrene decanoic acid can be used to quantify membrane fluidity. Briefly, WT and _EpsM_TcpH cells were subcultured from overnight cultures to an optical density of 0.01 in 100 ml of LB pH 6.5 in a 250 ml Erlenmeyer flask. *V. cholerae* strains were grown for 4 hours under Vir Ind conditions, centrifuged (2450 X g 15 minutes), resuspended in 0.8 ml LB. 200 µl of resuspended cells were transferred to 50 ml of fresh Vir Ind media supplemented with ethanol (3%w/v), benzyl alcohol (20mM), DMSO (1% w/v), or α-linolenic acid (500 µM) in 125 ml erlenmeyer flasks. Cultures were incubated for an additional 4 hours under Vir Ind or Non-Vir Ind conditions. After incubation cells were collected from 1 ml of culture via centrifugation (2450 X g 15 minutes) and resuspended in 500 µl LB. Cells were incubated with the fluorescent lipid reagent (10 µM final concentration) for 30 minutes at room temperature (~23ºC) while shaking. Cells were then washed twice with LB and fluorescence (excitation, 350 nm, and emission, 400 nm and 470 nm) was quantified for each sample. After subtracting the background fluorescence, the fluorescence ratio was calculated for each sample by dividing the excimer (470 nm) by the monomer (400 nm) fluorescence. Unlabeled cells and non-Vir ind conditions were used as negative controls. Ethanol and benzyl alcohol were used as positive controls.

**Mass Spectrometry**

Sample fatty acid and metabolite extraction was performed by protein precipitation with ethanol, as previously described [(6)](about:blank). To each sample, 20 nanograms of d8-arachidonic acid was added. Samples were homogenized in a ThermoFisher bead mill for 2 minutes. Homogenates were transferred to 2.0 mL centrifuge tubes and vortexed for 30 minutes, then incubated at -20C for one hour to precipitate proteins. Samples were then centrifuged at 15,000xg for 20 minutes. The supernatants were transferred to new 2.0 mL centrifuge tubes, and the remaining protein pellets were re-extracted and the supernatants pooled with those from the first extraction. Samples were dried under vacuum in a speedvac centrifuge, reconstituted in 200 microliters of acetonitrile, and transferred to LC-MS vials containing small volume inserts. Samples were stored at -80C until analysis. The LC-MS platform consisted of a Shimadzu Prominence HPLC coupled to a Thermo LTQ-Orbitrap Velos mass spectrometer. The LC system included two LC20AD pumps, a vacuum degassing system, autosampler, and column oven. The HPLC column was a Phenomenex 2.0 mmx150 mm Synergi HydroRP-C18 (4 micron, 80Angstrom pore size) equipped with a guard cartridge of the same column chemistry. The LC gradient was adapted from Watrous et al., Directed Non-targeted Mass Spectrometry and Chemical Networking for Discovery of Eicosanoids and Related Oxylipins, Cell Chemical Biology (2018), https://doi.org/10.1016/j.chembiol.2018.11.015. Solvent A was 70:30 water:acetonitrile (v:v) containing 0.1% acetic acid. Solvent B was 50:50 isopropanol:acetonitrile containing 0.02% acetic acid. Solvent C was isopropanol containing 0.02% acetic acid. The flow rate was 200 microliters per minute and the column oven was held at 45 degrees C. The autosampler was held at 4 degrees C. 10 microliters of each sample was injected. The gradient conditions used were: Time 0-2 minutes, 1% solvent B and 0% solvent C. Column eluent was diverted to waste using a 2-position 6 port valve. At time=2.0 minutes, Solvent B was increased to 50% with 0% solvent C, and a linear gradient from 50% to 65% B was run between 2.0 and 10 minutes while solvent C remained 0%. Solvent B then increased linearly to 87% B between 10 and 14 minutes with solvent C at 0%. Solvent C then was increased from 87% to 99% from 14-16 minutes while solvent B was dropped to 0%. Solvent C was then held constant at 99% until 24 minutes. Solvent C was then returned to 0% and solvent B was returned to 1% to re-equilibrate the column for 5 minutes. Column eluent was introduced to a Thermo LTQ-Orbitrap Velos mass spectrometer via a heated electrospray ionization source. The mass spectrometer was operated in negative ion mode at 60,000 resolution with full scan MS data collected from 200-700 m/z. Data-dependant product ion spectra were collected on the 4 most abundant ions at 7,500 resolution using the FT analyzer. The electrospray ionization source was maintained at a spray voltage of 4.5kV with sheath gas at 30 (arbitrary units) and auxiliary gas at 10 (arbitrary units). The inlet of the mass spectrometer was held at 350 degrees C, and the S-lens was set to 35%. The heated ESI source was maintained at 350 degrees Celsius. Chromatographic alignment, isotope correction, peak identification and peak area calculations were performed using MAVEN software. Concentrations of each analyte were determined against the peak area of the d8-arachidonic acid internal standard. Confirmed analytes were identified by comparison of aligned LC-MS peaks to the median m/z values, retention times (RT), and MS/MS fragmentation spectra of authentic reference standards. Additional fatty acids (C24-C28) were identified by comparison against certified reference materials and natural products as no reference standards are readily available.

| **Table S1: Fatty acids detected in lumen of infant mouse gastrointestinal tract.** Data presented here were collected from pooled infant mouse lumen. Lumen from three infant mice were pooled into one sample, and each sample was measured three times. Three pooled samples, from a total of nine infant mice, were collected. Data presented here were collected by the MSU Mass Spectrometry Core. | | | | |
| --- | --- | --- | --- | --- |
| **Compound** | **median m/z** | **median RT** | **Average (pg/ml)** | **Standard Deviation** |
| C14:0FA | 227.2011 | 12.6092 | 4458011.698 | 2135236 |
| C15:1 FA | 239.2016 | 11.32873 | 7134.024081 | 2377.688 |
| C15:0 FA | 241.2171 | 14.02128 | 638354.601 | 60356.9 |
| C16:1FA | 253.2169 | 13.04026 | 4893712.829 | 1976420 |
| C16:0FA | 255.2324 | 15.05075 | 11425307.99 | 2413736 |
| C17:1 FA | 267.233 | 14.27269 | 479804.594 | 232725.9 |
| C17:0 FA | 269.2486 | 15.89911 | 963601.9876 | 108067.4 |
| C18:3FA | 277.2171 | 11.89361 | 1210190.962 | 629231.4 |
| C18:2FA | 279.2325 | 13.70887 | 15640700.24 | 5935869 |
| C18:1FA | 281.248 | 15.20121 | 18628900.73 | 3480197 |
| C18:0FA | 283.2637 | 16.5997 | 5073779.937 | 941826.4 |
| C19:1 FA | 295.264 | 15.96052 | 362067.9852 | 194939.6 |
| C19:0 FA | 297.2798 | 17.16665 | 593058.308 | 79217.27 |
| C20:5FA | 301.2176 | 11.43768 | 266229.4372 | 139272.4 |
| C20:4FA | 303.2327 | 13.30841 | 2714314.669 | 1368729 |
| C20:3FA | 305.2485 | 14.31498 | 2023166.295 | 1162933 |
| C21:1 FA | 323.2956 | 17.12333 | 22416.91667 | 7268.663 |
| C21:0 FA | 325.3112 | 17.71526 | 301147.8624 | 24078.71 |
| C22:6FA | 327.2325 | 12.79087 | 734987.1558 | 453270.9 |
| C22:5FA | 329.2485 | 13.63419 | 405309.4299 | 311524.8 |
| C22:4FA | 331.2639 | 14.84863 | 1034993.569 | 655953.2 |
| 5(S)-HpETE | 335.2199 | 12.9949 | 23277.15085 | 1923.441 |
| (+)17-HDHA | 343.228 | 12.76887 | 528.8230532 | 477.9533 |
| C23:1 FA | 351.327 | 17.69901 | 35835.13889 | 3395.71 |
| C23:0FA | 353.3427 | 17.88094 | 395870.8751 | 42374.36 |
| C24:6 FA | 355.2643 | 14.30131 | 100845.444 | 66428.33 |
| C24:5 FA | 357.2796 | 15.31865 | 76921.17919 | 52300.63 |
| C24:4 FA | 359.2955 | 16.05526 | 157170.8308 | 100725.1 |
| C24:1FA | 365.3427 | 17.82233 | 370831.0044 | 102376.8 |
| C24:6hydroxy FA | 371.2591 | 12.1561 | 642.2208817 | 118.5921 |
| C25:1 FA | 379.3584 | 17.9402 | 40044.36505 | 2517.325 |
| C25:0 FA | 381.374 | 18.10494 | 434018.0396 | 60942.05 |
| C26:6 FA | 383.2954 | 15.53299 | 17247.10071 | 12527.83 |
| C26:5 FA | 385.3113 | 16.32854 | 32062.97911 | 22475.17 |
| C24:6dihydroxy FA | 387.2519 | 14.33381 | 25340.83136 | 3493.845 |
| C26:4 FA | 387.3269 | 17.02779 | 9600.568234 | 5977.7 |
| N-Oleoyl Taurine | 388.2521 | 13.94157 | 11474.338 | 3575.605 |
| C24:5dihydroxy FA | 389.2686 | 15.46758 | 6915.612851 | 656.1469 |
| C26:1 FA | 393.3742 | 18.04401 | 85711.02744 | 14536.83 |
| N-Oleoyl Leucine | 394.3326 | 14.86783 | 268.6841632 | 143.8705 |
| C26:0 FA | 395.3896 | 18.17179 | 422825.2018 | 59608.89 |
| N-Oleoyl Glutamine | 409.3073 | 10.22164 | 91.20159285 | 82.80444 |
| C28:5 FA | 413.3428 | 17.15413 | 462.1180753 | 213.9866 |
| C26:6dihydroxy FA | 415.2828 | 15.76583 | 542.5859934 | 290.3087 |
| C28:1 FA | 421.4056 | 18.33662 | 26726.6903 | 1766.721 |
| C28:0 FA | 423.4211 | 18.33743 | 422342.6307 | 30352.1 |
| C30:6 FA | 439.3586 | 17.27328 | 229.6001405 | 100.219 |
| C30:4 FA | 443.39 | 17.47948 | 242.3905897 | 136.996 |
| C30:1 FA | 449.4369 | 18.48088 | 20098.28803 | 1848.832 |
| C30 FA | 451.4524 | 18.4765 | 293145.4574 | 28012.25 |
| C32:1 FA | 477.4682 | 18.53508 | 13902.54468 | 851.3785 |
| C32:0 FA | 479.4837 | 18.60744 | 169780.3186 | 16506.48 |
| C32:4hydroxyFA | 487.4161 | 17.48545 | 251.5879774 | 105.8449 |
| C32:5dihydroxy FA | 501.3988 | 17.79052 | 401.913033 | 212.0956 |
| C34:3 FA | 501.4681 | 18.51305 | 208.3844935 | 67.00445 |
| C32:4dihydorxy FA | 503.4079 | 12.92139 | 1398.878109 | 1002.818 |
| C34:2FA | 503.484 | 18.5617 | 729.9102596 | 101.7631 |
| C34:1 FA | 505.4995 | 18.6485 | 10064.12756 | 888.0155 |
| C34:0 FA | 507.515 | 18.73401 | 127059.502 | 16923.4 |
| C34:6dihydroxy FA | 527.4082 | 12.50761 | 883.0972963 | 731.6948 |
| C36:5dihydroxy FA | 529.4234 | 13.02639 | 67514.76292 | 20026.99 |
| C38:5 FA | 553.5032 | 17.8609 | 122.77066 | 90.27743 |

**Table S2:** strains used in this study.

| **Strain** | **Description** | **Reference** |
| --- | --- | --- |
| *V. cholerae* 0395 classical biotype | Wild type | DiRita lab collection |
| *V. cholerae ∆tcpH* | Isogenic deletion | DiRita lab collection |
| *V. cholerae ∆tcpP* | Isogenic deletion | DiRita lab collection |
| *V. cholerae ∆tcpH* pBAD18-empty vector | Overexpression plasmid vector | DiRita lab collection |
| *V. cholerae ∆tcpH* pBAD18 TcpH | *∆tcpH* complementation with ectopic *tcpH* | This study |
| *V. cholerae ∆tcpPH* pBAD18 _CtxB_TcpH | *∆tcpH* complementation with ectopic *tcpH* TM construct | This study |
| *V. cholerae ∆tcpH*; *∆yaeL* pBAD18-empty vector | Overexpression plasmid | This study |
| *V. cholerae ∆tcpH*; *∆yaeL* pBAD18 _CtxB_TcpH | *∆tcpH* complementation with ectopic *tcpH* TM construct | This study |
| *V. cholerae ∆tcpH*; *∆yaeL* pBAD18 _ToxS_TcpH | *∆tcpH* complementation with ectopic *tcpH* TM construct | This study |
| *V. cholerae ∆tcpH*; *∆yaeL* pBAD18 _EpsM_TcpH | *∆tcpH* complementation with ectopic *tcpH* TM construct | This study |
| *V. cholerae ∆tcpH*; *∆yaeL*  pBAD18 TcpH_∆136-119_ | *∆tcpH* complementation with ectopic *tcpH* Peri construct | This study |
| *V. cholerae ∆tcpH*; *∆yaeL*  pBAD18 TcpH_∆136-103_ | *∆tcpH* complementation with ectopic *tcpH* Peri construct | This study |
| *V. cholerae ∆tcpP*  pBAD18 *Hsv-His(6x)-tcpP* | N-terminal *tcpP* co-immuno precipitation construct | This study |
| *V. cholerae ∆tcpP* pBAD18 *tcpP-His(6x)-Hsv* | C-terminal *tcpP* co-immuno precipitation construct | This study |
| *V. cholerae ∆tcpH* pBAD18 *Hsv-His(6x)-tcpH* | N-terminal *tcpH* co-immuno precipitation construct | This study |
| *V. cholerae ∆tcpH* pBAD18 *tcpH-His(6x)-Hsv* | C-terminal *tcpH* co-immuno precipitation construct | This study |
| *V. cholerae ∆yaeL*  pBAD18 *Hsv-His(6x)-tcpP* | N-terminal *tcpP* co-immuno precipitation construct | This study |
| *V. cholerae ∆yaeL* pBAD18 *tcpP-His(6x)-Hsv* | C-terminal *tcpP* co-immuno precipitation construct | This study |
| *V. cholerae* _CtxB_TcpH | chromosomal construct | This study |
| *V. cholerae* _ToxS_TcpH | chromosomal construct | This study |
| *V. cholerae* _EpsM_TcpH | chromosomal construct | This study |
| *V. cholerae* TcpH_∆136-119_ | chromosomal construct | This study |
| *V. cholerae* TcpH_∆136-103_ | chromosomal construct | This study |
| *V. cholerae* TcpH_∆119-103_ | chromosomal construct | This study |
| *V. cholerae* TcpH_∆103-79_ | chromosomal construct | This study |
| *V. cholerae* TcpH_∆79-55_ | chromosomal construct | This study |
| *V. cholerae* TcpHC114S | isogenic mutant | This study |
| *V. cholerae* TcpHC114S/C132S | isogenic mutant | This study |
| *V. cholerae* pBH6119-*toxT::GFP* | *toxT* transcription reporter | Anthouard R, and DiRita VJ. mBio. 2013. |
| *V. cholerae ∆tcpH* pBH6119-*toxT::GFP* | *toxT* transcription reporter | This study |
| *V. cholerae ∆tcpP* pBH6119-*toxT::GFP* | *toxT* transcription reporter | This study |
| *V. cholerae* _CtxB_TcpH pBH6119-*toxT::GFP* | *toxT* transcription reporter | This study |
| *V. cholerae* _ToxS_TcpH pBH6119-*toxT::GFP* | *toxT* transcription reporter | This study |
| *V. cholerae* _EpsM_TcpH pBH6119-*toxT::GFP* | *toxT* transcription reporter | This study |
| *V. cholerae* TcpH_∆136-119_ pBH6119-*toxT::GFP* | *toxT* transcription reporter | This study |
| *V. cholerae* TcpH_∆136-103_ pBH6119-*toxT::GFP* | *toxT* transcription reporter | This study |
| *V. cholerae* TcpH_∆119-103_ pBH6119-*toxT::GFP* | *toxT* transcription reporter | This study |
| *V. cholerae* TcpH_∆103-79_ pBH6119-*toxT::GFP* | *toxT* transcription reporter | This study |
| *V. cholerae* TcpH_∆79-55_ pBH6119-*toxT::GFP* | *toxT* transcription reporter | This study |
| *V. cholerae* TcpHC114S pBH6119-*toxT::GFP* | *toxT* transcription reporter | This study |
| *V. cholerae* TcpHC114S/C132S pBH6119-*toxT::GFP* | *toxT* transcription reporter | This study |
| *E. coli* ET12567  *∆dapA* | Cloning vector recipient | Allard, N., et. al. 2015. Canadian Journal of Microbiology, 61(8), pp.565-574. |
| *E. coli* ET12567 *∆dapA* pKAS32-empty vector | Plasmid vector strain | DiRita lab collection |
| *E. coli* ET12567 *∆dapA* pBAD18-empty vector | Plasmid vector strain | DiRita lab collection |

**Table S3:** All primers used in this study contain Kpn1-HiFi (forward primers) and Xba1 (reverse primers) restriction sites.

| **Description** | **Sequence** |
| --- | --- |
| pKAS FW | gcctctaaggttttaagt |
| pKAS RV | ctttcaaggtagcggttacc |
| pBAD18 FW | ctgtttctccatacccgtt |
| pBAD18 RV | ggctgaaaatcttctct |
| pKAS-TcpP promoter FW | ctaacgttaacaaccggtactttcgagtgatagaaaaagg |
| pKAS-TcpP FW | ctaacgttaacaaccggtacatggggtatgtccgcgtg |
| pKAS-downstream TcpH RV | aaatttgcgcatgctagctatagttcttggtcttttttagataacgtaagc |
| TcpP-CtxBss FW | atgcactaaaaattaaaagacattagaatgattaaattaaaatttgg |
| TcpP-CtxBss RV | aatttaatcattctaatgtcttttaatttttagtgcattctaatgtcttc |
| CtxBss-TcpHperi FW | tcttcagcatatgcacatggaccgatgcgacaaaaaaac |
| CtxBss-TcpHperi RV | gtcgcatcggtccatgtgcatatgctgaaga |
| TcpP-EpsMss FW | atgcactaaaaattaaaagacattagaatgatgaaagaattattggctc |
| TcpP-EpsMss RV | tctaatgtcttttaatttttagtgcattctaatgtcttc |
| EpsMss-TcpHperi FW | gggaatatggccgatgcgacaaaaaaac |
| EpsMss-TcpHperi RV | gtcgcatcggccatattccccaataagc |
| TcpP-ToxSss FW | atgcactaaaaattaaaagacattagaatgcaaaatagacacatcg |
| TcpP-ToxSss RV | cgatgtgtctattttgcattctaatgtcttttaatttttagtgcattctaatgtcttc |
| ToxSss-TcpHperi FW | ttgggggagtccgatgcgacaaaaaaac |
| ToxSss-TcpHperi RV | tgcatgcctgcaggtcgactctaaaaatcgctttgacag |
| TcpH_∆136-119_ FW | cgccttcccttagggtcttatcatgagccgc |
| TcpH_∆136-119_ RV | tgataagaccctaagggaaggcgagaaaacaac |
| TcpH_∆136-103_ FW | tgattacaattagggtcttatcatgagccgc |
| TcpH_∆136-103_ RV | tgataagaccctaattgtaatcacggctcacattactttc |
| TcpH_∆119-103_ FW | tgattacaattacaagcagcttacggctg |
| TcpH_∆119-103_ RV | taagctgcttgtaattgtaatcacggctcac |
| TcpH_∆103-79_ FW | tcaaacattggtgttgagtatttatcaactc |
| TcpH_∆103-79_ RV | tactcaacaccaatgtttgataacgtgtag |
| TcpH_∆79-55_ FW | taatctatccccagatcctagctctcag |
| TcpH_∆79-55_ RV | taggatctggggatagattaccttgataagtag |
| TcpHC114S FW | tcaactcggcaaaggtagttttctcgccttccc |
| TcpHC114S RV | gggaaggcgagaaaactacctttgccgagttga |
| TcpHC132S FW | ggttttccagtcaaagcgatttttag |
| TcpHC132S RV | ctaaaaatcgctttgactggaaaacc |
| pBAD18-CtxBss FW | agcgaattcgagctcggtaccaaagggagcattataagacattagaatgattaaattaaaatttgg |
| pBAD18-ToxSss RV | agcgaattcgagctcggtaccaaagggagcattatatgcaaaatagacacatcg |
| pBAD18-EpsMss FW | agcgaattcgagctcggtaccaaagggagcattatatgatgaaagaattattggctc |
| pBAD18-TcpH FW | agcgaattcgagctcggtaccaaagggagcattatatgcacaaaaaattaaaagcttg |
| pBAD18-TcpH RV | tgcatgcctgcaggtcgactctaaaaatcgctttgacag |
| pBAD18-TcpH_∆136-119_ RV | tgcatgcctgcaggtcgactctaagggaaggcgagaaaacaac |
| pBAD18-TcpH_∆136-103_ RV | tgcatgcctgcaggtcgactctaattgtaatcacggctcacattactttc |
| pBAD18 Hsv-His(6x) FW | ttcgagctcggtaccaaagggagcattatatgcagccggaactggcgccggaagatccg |
| Hsv-His(6x)-TcpP FW | ccggaagatccggaagattgccatcatcatcatcatcatatggggtatgtccgcgtg |
| Hsv-His(6x)-TcpP RV | cagttccggctgatgatgatgatgatgatgattttttgtgcattctaatgtcttc |
| pBAD18-TcpP RV | tgcatgcctgcaggtcgactttaattttttgtgcattctaatgtcttctgttc |
| pKT25-TcpP FW | ggctgcagggtcgactatggggtatgtccgc |
| pKT25-TcpP RV | attcttacttacttaggtacttaattttttgtgcattctaatgtcttctgttc |
| pUT18C-TcpH FW | aacgccactgcaggtcgactcagcggtggtggaggttcgaaatgcacaaaaaattaaaag |
| pUT18C-TcpH RV | gatgaattcgagctcggtacctaaaaatcgctttgacaggaaaacc |
| *recA* FW RT-qPCR | attgaaggcgaaatgggcgatag |
| *recA* RV RT-qPCR | tacacatacagttggattgcttg agg |
| *toxT* FW RT-qPCR | actgatgatcttgatgctatggag |
| *toxT* RV RT-qPCR | catccgattcgttcttaattcacc |
| *tcpP* FW RT-qPCR | tgagtgggggaagataaacg |
| *tcpP* RV RT-qPCR | ttggattgttatccccggta |

**Supplemental Figures**

**Figure S1. Growth rates of Transmembrane (TM) and Periplasmic (Peri) TcpH variants are similar to WT cells. Related to Figures 1-3.** A) Virulence inducing conditions growth curve of TcpH TM and Peri constructs respectively. B) *in vitro* characterization of TcpH TM and Peri chromosomal constructs grown under virulence inducing conditions. Western blots of whole-cell lysates probed with α-TcpP (top), α-TcpH (middle), and α-TcpA (bottom). In addition, CtxB levels and *toxT* transcription, relative to WT, were also determined for the TcpH TM and Peri constructs. Average CtxB levels and *toxT* fold change (relative to WT) for each strain are indicated below the western blot. Data presented here were collected from three independent experiments. C) Virulence inducing condition growth curve supplemented with crude bile (0.4%). D) Virulence inducing condition growth curve supplemented with purified bile salts (cholate/deoxycholate 100µM). E) Virulence inducing condition growth curve supplemented with linolenic acid (500µM). F) LB, 37°C, growth curve with 1mM to 100nM Miltefosine.  A, C-F) Data presented here are the average of three independent experiments.

**Figure S2: (left figure panel) TcpH transmembrane and periplasmic constructs display WT growth in adult mice feces, and TcpH transmembrane constructs inhibit RIP of TcpP. Related to Figure 2.** A) Western blot of Initial inoculums used to infect infant mice in Figure 2A. B) End-point colony forming units during growth in filter sterilized mice fecal media. C) End-point colony forming units during growth in non-filtered (i.e, non-sterile) mice fecal media. Δ*tcpP* was excluded from non-sterile mice fecal growth experiment due to limited supply of non-sterile mice fecal media. D) Relative TcpA levels after 21hrs of aerobic growth in sterile adult mice fecal media (9% w/v). TcpA levels were determined via densitometry analysis of western blots, calculated using ImageJ. See Figure S10A for the western blot. B-D) Averages represent data collected from three or more independent experiments. Error bars represent standard error of the mean.

**Figure S3: (right figure panel) The transmembrane domain of TcpH is conserved across *V. cholerae* biotypes.** Multiple sequence alignment of the N-terminus of TcpH (the first 55 amino acids) from classical (0395) and non-classical V. cholerae strains. *: indicates the end of the transmembrane domain.

**Figure S4: α-Linolenic acid stimulates *toxT* transcription in a TcpH dependent manner. Related to Figure 3.** A) *toxT* transcription was determined using a plasmid based *toxT::GFP* transcriptional reporter. B) *toxT* transcription in WT *V. cholerae* cells using RT-qPCR, determined via ∆∆CT method. Cells were incubated in Vir Ind for 4hrs and then transferred to indicated conditions for an additional 4hrs. RNA was collected at the 8hr time point. C-D) *toxT* expression in WT cells determined using a plasmid based *toxT::GFP* transcription reporter. Concentrations of fatty acids used are displayed below each bar. A-D) Data were collected from three or more independent experiments. A-C) Error bars represent the standard error of the mean. D) Error bars represent standard deviation. A-D) A one-way ANOVA was used to determine statistical significance. *indicates a P-value of < 0.05.

**Figure S5: α-Linolenic acid is utilized by *V. cholerae* cells, α-linolenic increases membrane fluidity, and does not influence *tcpP* transcription, or promote non-specific protein association within detergent resistant membranes. Related to Figure 3 and 4.** A) *tcpP* transcription in WT *V. cholerae* cells determined using *tcpP::lacZ* transcription reporter. *tcpP* transcription was determined by quantifying LacZ activity (i.e., calculating Miller Units) [(7)](about:blank). Data was collected from five independent experiments. B) Molar percentage of fatty acids present in whole *V. cholerae* cells. LA, indicates cells were cultured with α-linolenic acid. Error bars represent the standard deviation, and the average values here were collected from two independent experiments. C) WT (black bars) and _EpsM_TcpH (gray bars) colony forming units after 24 hours of growth in M9 minimal media (0.05% glucose) at 37°C. The legend below indicates the presence of additional compounds or fatty acids (“+” indicates present and “-” indicates absence). DMSO and cerulenin final concentration 1% (v/v) and 10 µg/ml respectively. Data was collected from 4 independent experiments. Error bars indicate standard deviation. A one-way ANOVA was used to determine statistical significance. *indicates a P-value of < 0.05.

**Figure S6: Spheroplast TI/TS membrane extraction demonstrates that TcpP and TcpH increase in abundance in TI membrane fractions with α-linolenic acid.** A) Abundance of TcpP molecules within the Triton insoluble (TI, top western) Triton soluble (TS, bottom western) membranes in WT or _EpsM_TcpH cells growth with or without 500 µM α-linolenic acid (LA). B) Densitometry analysis of western blots in panel A. Densitometry analysis done using ImageJ. C) Relative abundance of TcpH within the TI and TS membrane fractions membranes in WT and _EpsM_TcpH cells growth with or without 500 µM α-linolenic acid. The black arrow indicates the TcpH specific bands. D) Densitometry analysis of westerns in panel C. Densitometry analysis done using ImageJ. B and D) Error bars represent the standard deviation. A one-way ANOVA was used to determine statistical significance. ns indicates a lack of statistical significance. A-D) Data presented here were collected from three independent experiments. TI and TS membrane fractions collected by gentle freeze thaw lysis.

**Figure S7: Arachidonic and Docosahexaenoic acid do not change the abundance of TcpP or TcpH in TI and TS membrane fractions.** A) Abundance of TcpP molecules within the Triton insoluble (TI, top western) Triton soluble (TS, bottom western) membranes in WT cells growth with or without 500 µM arachidonic (Ara500) or docosahexaenoic acid (Doco500). B) Densitometry analysis of western blots in panel A. C) Relative abundance of TcpH within the TI and TS membrane fractions membranes in WT cells growth with or without 500 µM arachidonic (Ara500) or docosahexaenoic acid (Doco500). D) Densitometry analysis of westerns in panel C. B and D) TcpP and TcpH abundance were measured via densitometry using ImageJ. Error bars represent the standard deviation. A one-way ANOVA was used to determine statistical significance. ns indicates a lack of statistical significance. A-D) Data presented here were collected from three independent experiments. TI and TS membrane fractions collected by gentle freeze thaw lysis.

**Figure S8: α-linolenic increases does not promote non-specific protein association within detergent resistant membranes, and Hsv-His(6x) tagged TcpP constructs remain functional. Related to Figure 4 and 5.** A) Relative levels of the non-specific loading control in α-TcpH westerns is equally distributed among Triton soluble (i.e., TS; lipid disordered) and Triton insoluble (i.e., TI; lipid ordered) fractions. Addition of α-linolenic acid (LA, 500µM), indicated by +/-, does not change this distribution. Relative levels of the non-specific loading control were determined via densitometry analysis. Densitometry analysis was conducted using ImageJ. Error bars represent the standard error of the mean. Data were collected from three independent experiments. See Figure S6 and Figure S10EF. B) Molar percentage of fatty acids within WT *V. cholerae* Triton soluble (i.e., TS; lipid disordered) and Triton insoluble (i.e., TI; lipid ordered) fractions. LA, indicates cells were cultured with α-linolenic acid. Error bars represent the standard deviation, and the average values here were collected from two independent experiments. C) CtxB levels, measured via ELISA, in culture supernatants collected from cultures incubated with *V. cholerae* cells cultured in virulence inducing conditions for 24hrs. Black bars represent WT cells. Light gray bars represent ∆*tcpP* complemented with pBAD18-*Hsv-His(6x)-tcpP* , and dark gray bars represent ∆ *tcpP* complemented with pBAD18-*tcpP-His(6x)-Hsv*. *tcpP* constructs were ectopically expressed from pBAD18 using arabinose (Ara 0.1% w/v). + indicates arabinose was added to the culture.

**Figure S9: α-linolenic acid promotes interaction between TcpP and TcpH within the TI membrane fraction.** A) TcpP-His-Hsv purified from Triton insoluble (TI, top western) membranes with or without 500 µM α-linolenic acid (LA). *tcpP-his-hsv* was ectopically expressed from pBAD18 using arabinose (Ara 0.1% w/v). + indicates arabinose was added to the culture. Samples probed with α-Hsv (top westerns, right western was overexposed to display lower abundance bands) and α-TcpH (bottom western). Proteins were crosslinked with suberic acid bis(N-hydroxysuccinimide ester) prior to cell lysis, and TI-2 buffer was used during these experiments. B) TcpH abundance in pull down fractions normalized to TcpP-His-Hsv abundance. Densitometry analysis of western blots in panel A. Densitometry analysis done using ImageJ. Error bars represent the standard deviation. A two-way Student's T-test was used to determine statistical significance. A-B) Data presented here were collected from three independent experiments. TI membrane fractions collected by gentle freeze thaw lysis. * Indicates that this TcpH band was no included in the analysis in panel B.

**Figure S10: Western blots used for densitometry analysis. Relevant to Figures 3, 4, S2, and S8.** A) TcpA levels in cells grown for 21 hours in mice fecal media. See Figure S2D for densitometry analysis. B) TcpP abundance in whole cell lysates in WT, _EpsM_TcpH, _TosX_TcpH, and Δ*tcpH* cells. See Figure 3B for densitometry analysis. C and D) TcpP abundance in TI (Triton insoluble) and TS (Triton soluble) membranes in WT and _EpsM_TcpH cells grown with or without α-linolenic acid (LA, 500µM). See Figure 4B for densitometry analysis. E and F) TcpH levels within TI and TS membranes in WT and _EpsM_TcpH cells grown with and without LA. See Figure 4C for densitometry analysis.

**Supplemental Material References**

1. [T. D. Schmittgen, K. J. Livak, Analyzing real-time PCR data by the comparative CT method. *Nature Protocols* **3**, 1101–1108 (2008).](about:blank)

2. [L. Jiang, L. He, M. Fountoulakis, Comparison of protein precipitation methods for sample preparation prior to proteomic analysis. *J. Chromatogr. A* **1023**, 317–320 (2004).](about:blank)

3. [S. Quan, A. Hiniker, J.-F. Collet, J. C. A. Bardwell, Isolation of bacteria envelope proteins. *Methods Mol. Biol.* **966**, 359–366 (2013).](about:blank)

4. [V. L. Miller, R. K. Taylor, J. J. Mekalanos, Cholera toxin transcriptional activator ToxR is a transmembrane DNA binding protein. *Cell* **48**, 271–279 (1987).](about:blank)

5. [Z. Wang, C. Benning, Arabidopsis thaliana polar glycerolipid profiling by thin layer chromatography (TLC) coupled with gas-liquid chromatography (GLC). *J. Vis. Exp.* (2011) https:/doi.org/10.3791/2518.](about:blank)

6. [J. D. Watrous, *et al.*, Directed Non-targeted Mass Spectrometry and Chemical Networking for Discovery of Eicosanoids and Related Oxylipins. *Cell Chem Biol* **26**, 433–442.e4 (2019).](about:blank)

7. [J. H. Miller, Cold Spring Harbor Laboratory, *Experiments in Molecular Genetics* (1974).](about:blank)
