# Supplementary figures and images for: An essential host dietary fatty acid promotes TcpH inhibition of TcpP proteolysis promoting virulence gene expression in Vibrio cholerae
Source: mBio. 2024 Jul 3;15(8):e00721-24. doi: 10.1128/mbio.00721-24 (PMC11323476; doi:10.1128/mbio.00721-24)

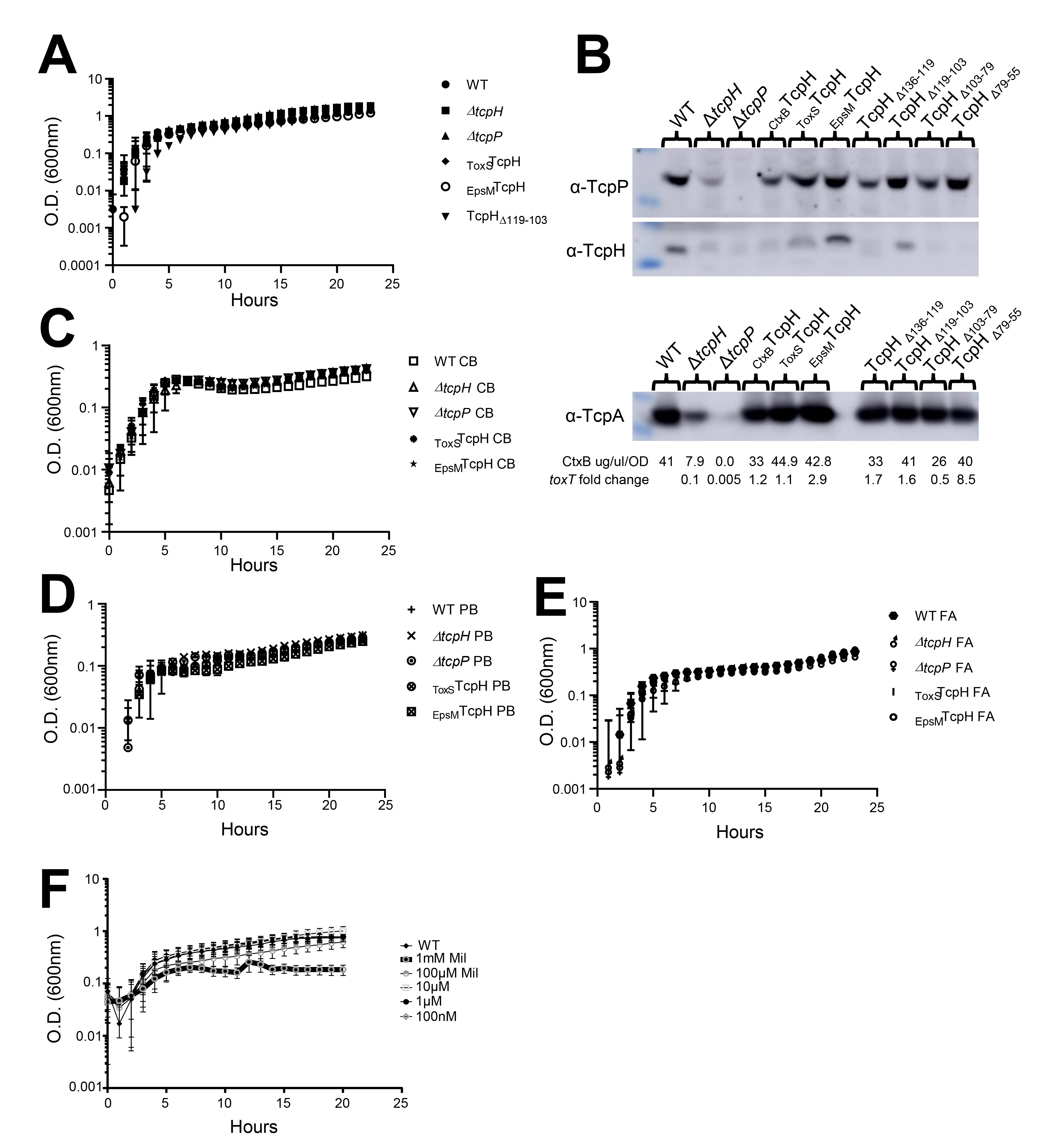

Supplement: Figure S1 — Growth rates of transmembrane and periplasmic TcpH variants are similar to WT cells. [file mbio.00721-24-s0002.tif]

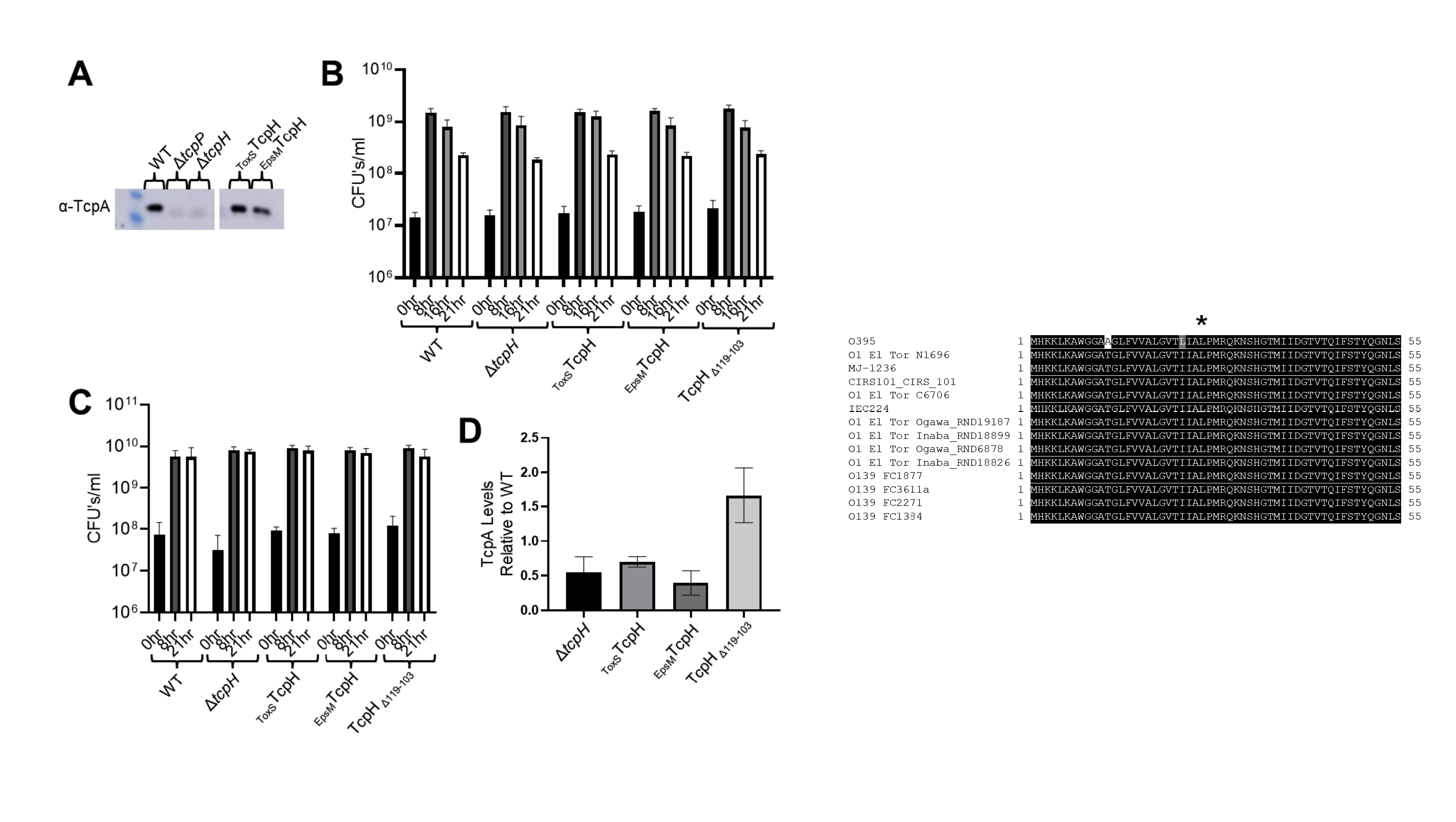

Supplement: Figures S2 and S3 — Constructs in adult mice and conservation of TM domain. [file mbio.00721-24-s0003.tiff]

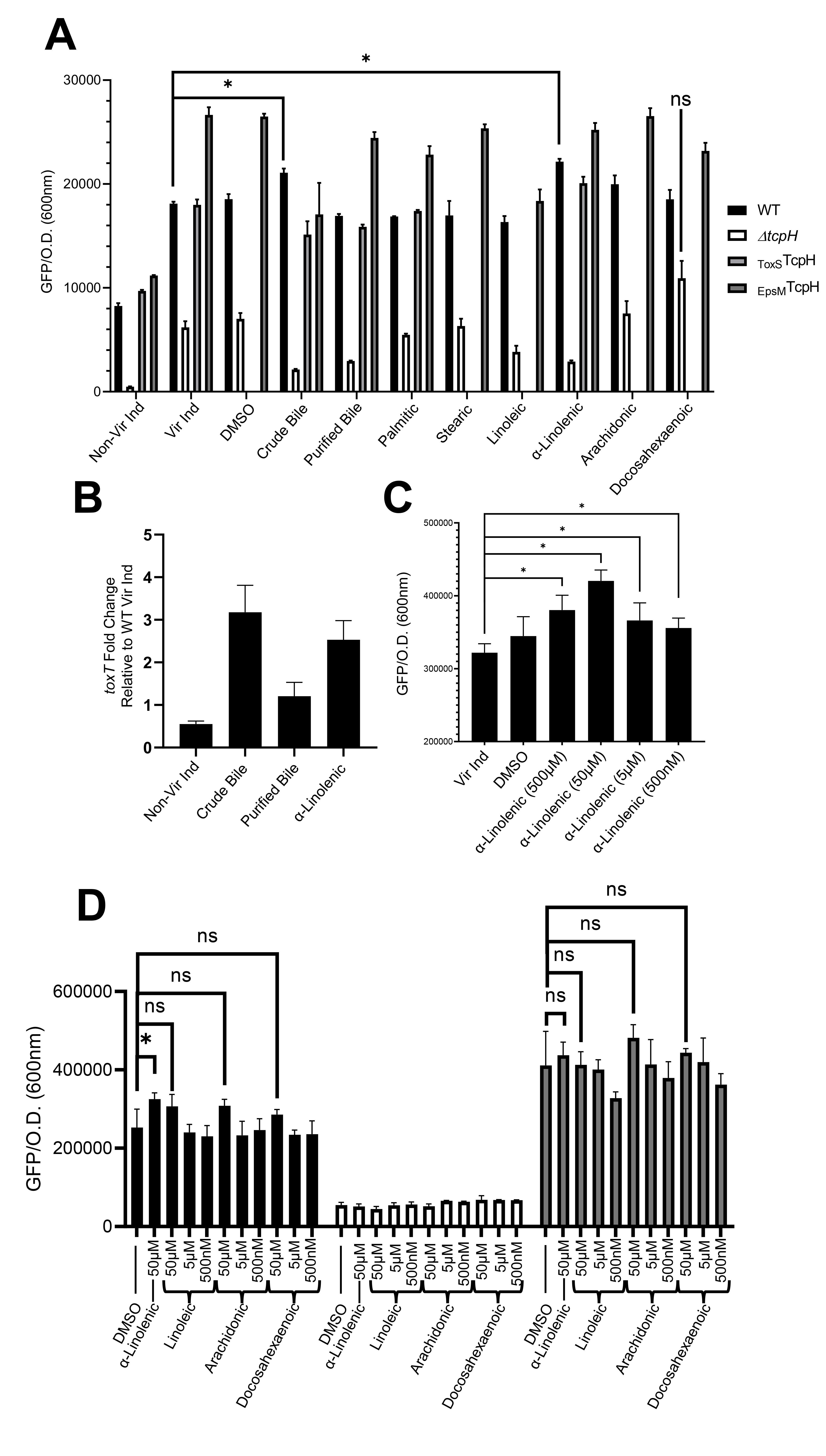

Supplement: Figure S4 — α-Linolenic acid stimulates toxT transcription in a TcpH-dependent manner. [file mbio.00721-24-s0004.tif]

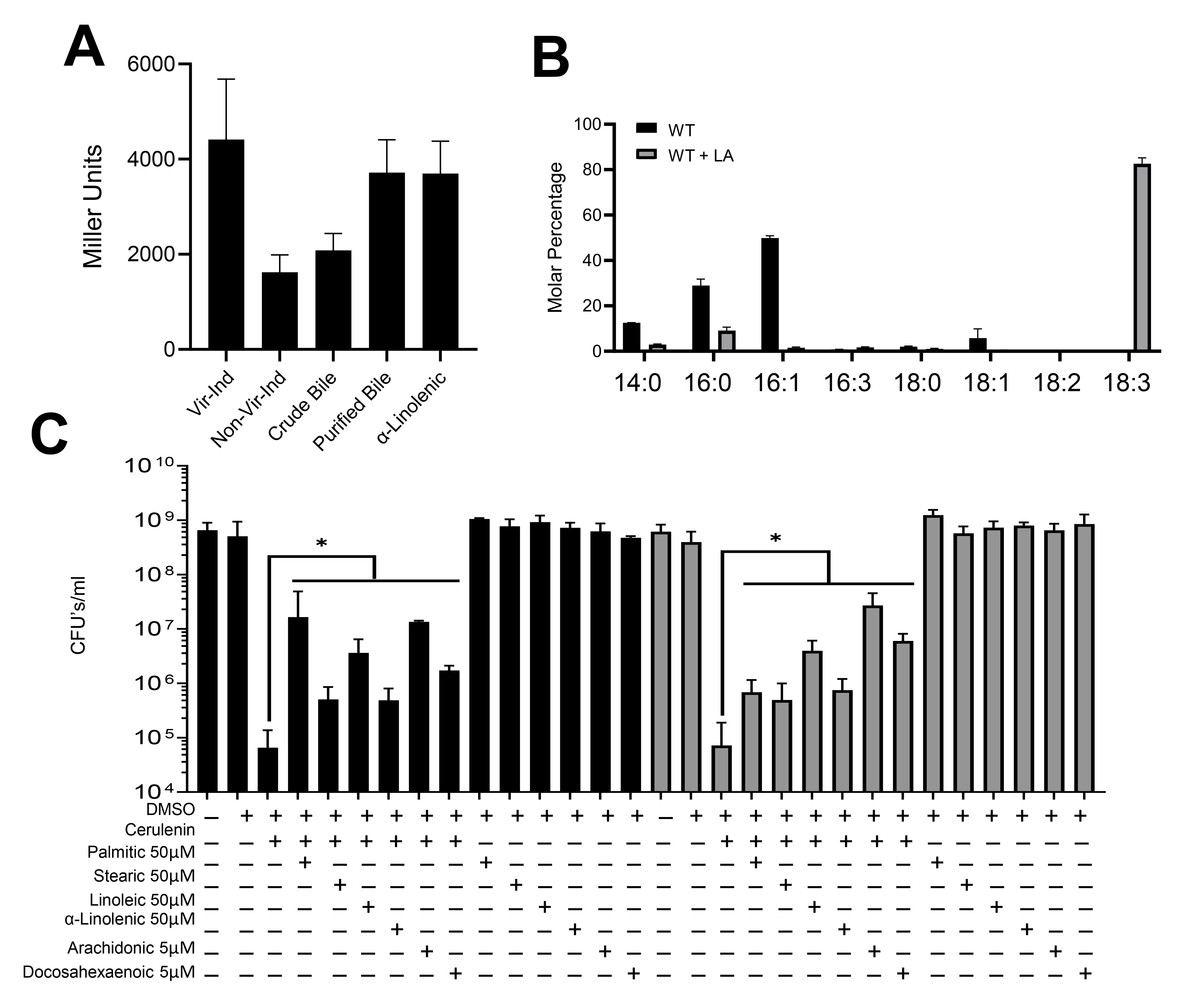

Supplement: Figure S5 — α-Linolenic acid is utilized by V. cholerae cells, increases membrane fluidity, and does not influence tcpP transcription or promote non-specific protein association within detergent-resistant membranes. [file mbio.00721-24-s0005.tif]

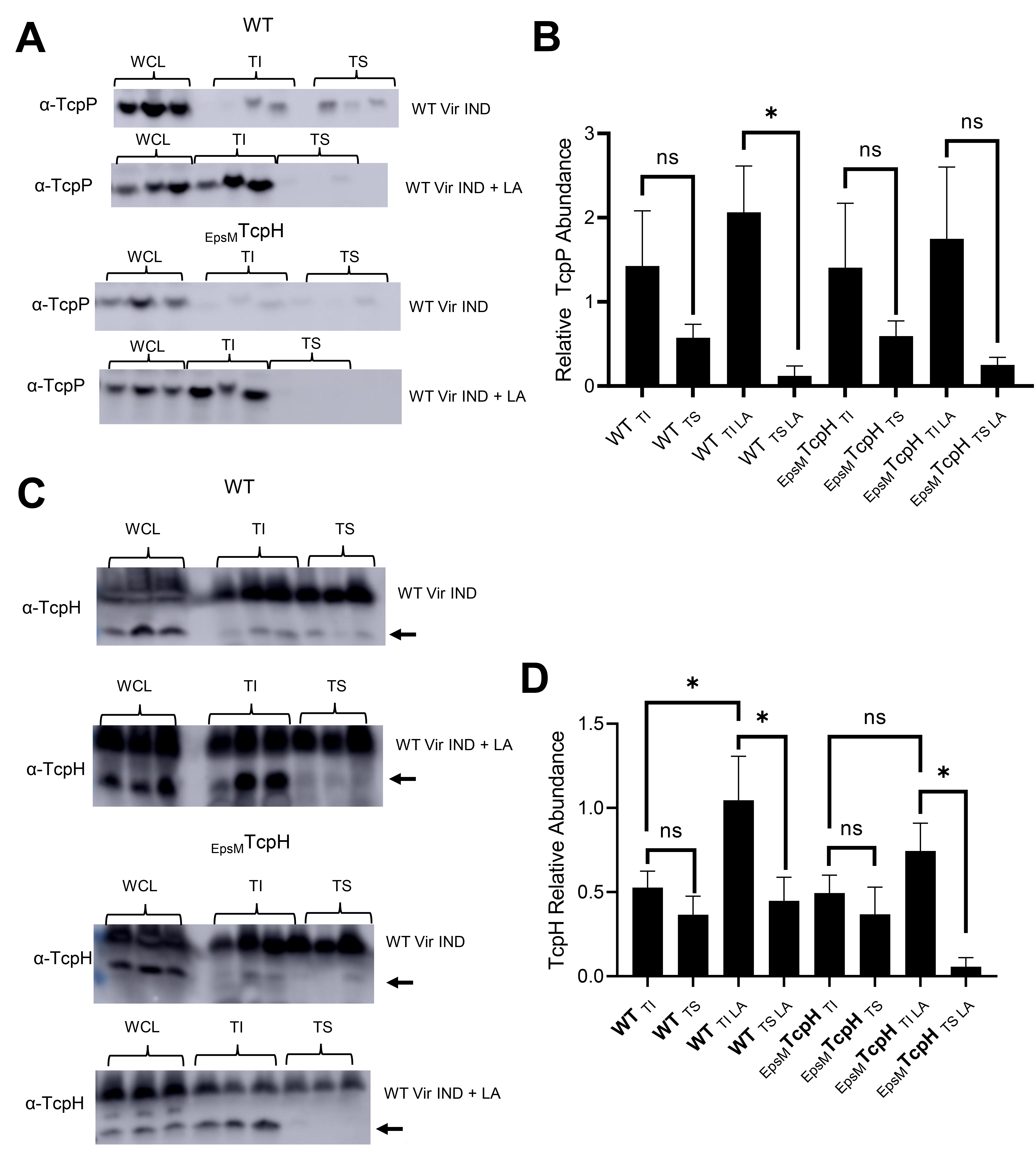

Supplement: Figure S6 — Spheroplast TI/TS membrane extraction demonstrates that TcpP and TcpH increase in abundance in TI membrane fractions with α-linolenic acid. [file mbio.00721-24-s0006.tif]

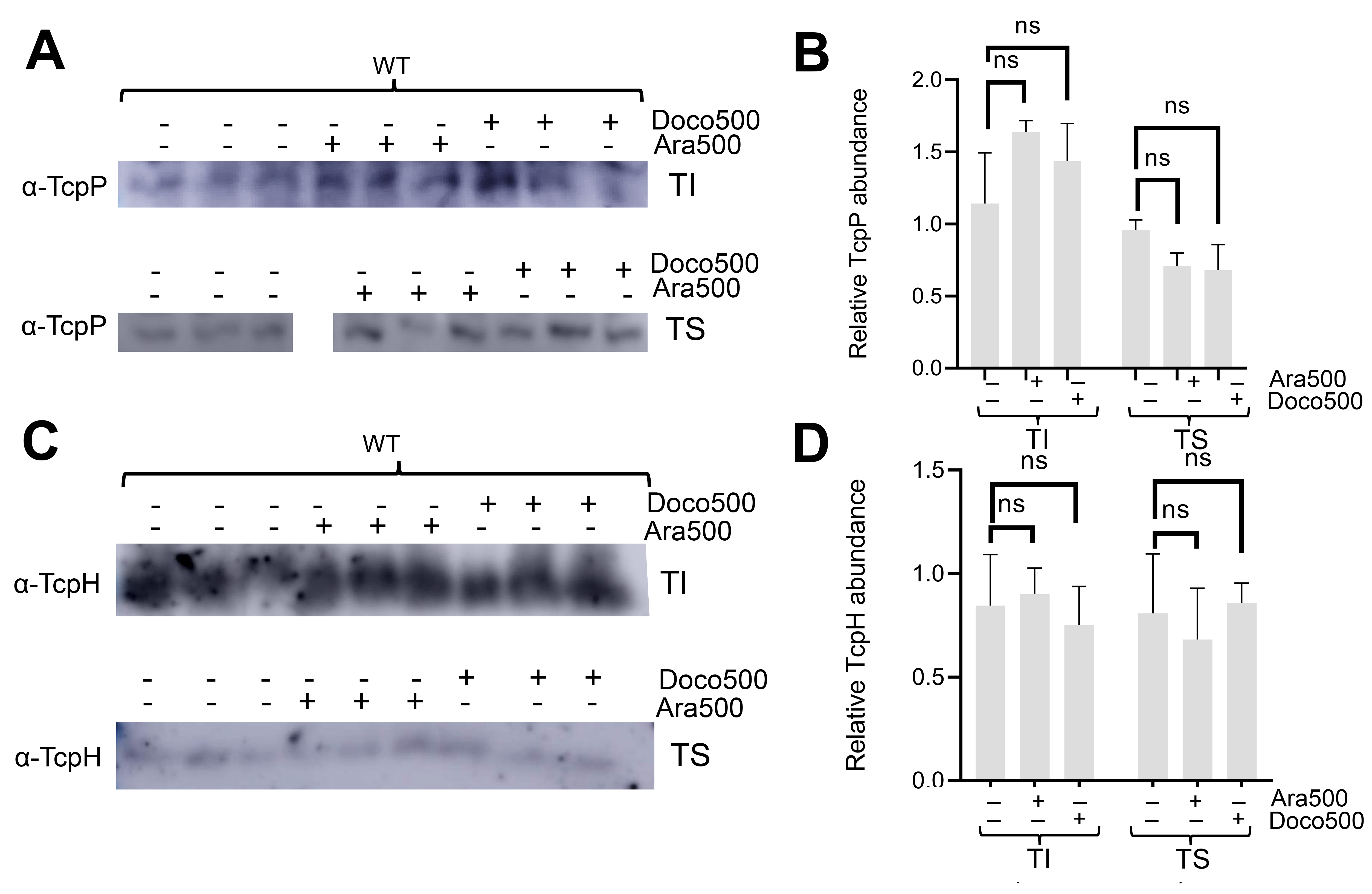

Supplement: Figure S7 — Arachidonic and docosahexaenoic acid do not change the abundance of TcpP or TcpH in TI and TS membrane fractions. [file mbio.00721-24-s0007.tif]

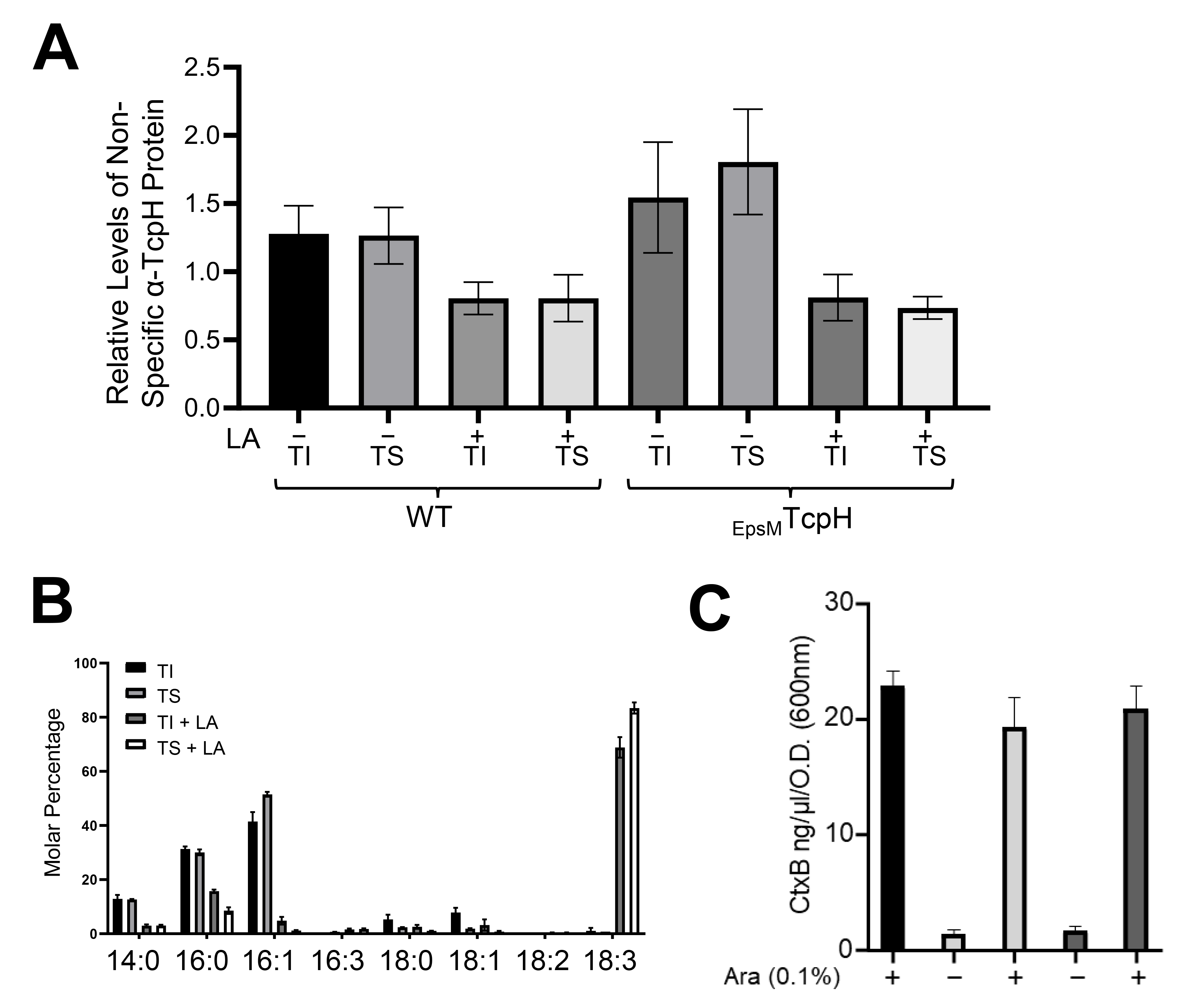

Supplement: Figure S8 — α-Linolenic acid does not promote non-specific protein association within detergent-resistant membranes, and Hsv-His(6×)-tagged TcpP constructs remain functional. [file mbio.00721-24-s0008.tif]

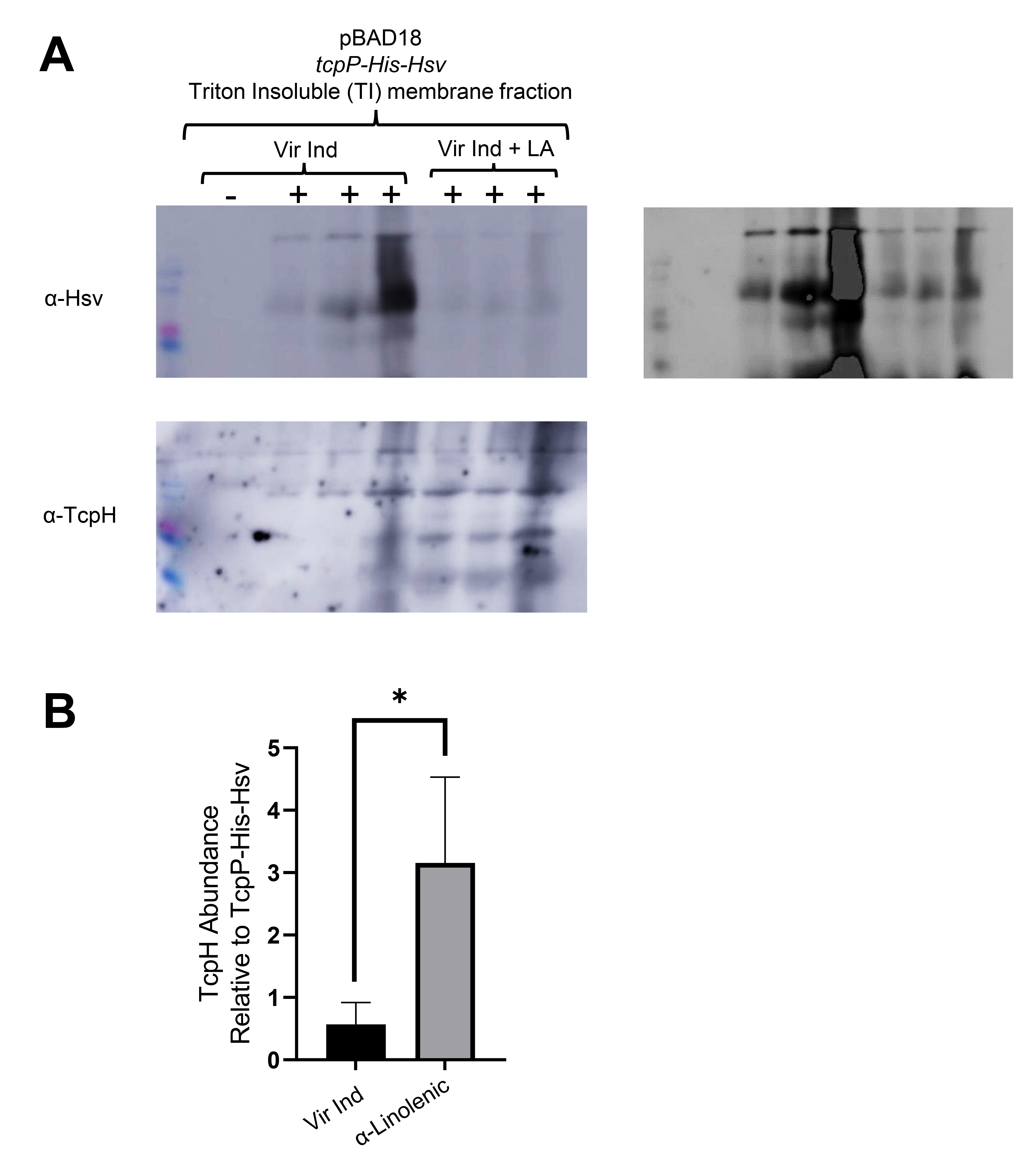

Supplement: Figure S9 — α-Linolenic acid promotes interaction between TcpP and TcpH within the TI membrane fraction. [file mbio.00721-24-s0009.tif]
